# Identification of Four Compounds with S-RBD-Binding and Pseudovirus Entry-Inhibitory Activity

**DOI:** 10.3390/ijms27188136

**Published:** 2026-09-12

**Authors:** Jingjie Zheng, Shitao Wang, Yuqing Zhou, Qiman Lin, Jingsong Guan, Yao Wang, Jianglin Fan

**Affiliations:** 1 Guangdong Provincial Key Laboratory of Large Animal Models for Biomedicine, South China Institute of Large Animal Models for Biomedicine, School of Pharmacy and Food Engineering, Wuyi University, Jiangmen 529020, China; jingjie318@163.com (J.Z.); wangshitaofj@163.com (S.W.); 18824286742@163.com (Y.Z.); lam_1232024@163.com (Q.L.); 2112207098@wyu.edu.cn (J.G.); 2 Engineering Research Center in Ministry of Education for Innovative Drugs of Traditional Chinese Medicine and Zhuang Yao Medicine, Guangxi University of Chinese Medicine, Nanning 530001, China

**Keywords:** SARS-CoV-2 variants, spike receptor-binding domain, molecular docking, pseudovirus entry inhibition

## Abstract

COVID-19, caused by SARS-CoV-2, remains a global health challenge because of viral evolution and immune escape. Although current therapies primarily target viral entry and replication, agents that directly interfere with the Spike receptor-binding domain (S-RBD) remain limited. Here, we combined virtual screening with biological validation to identify compounds capable of interfering with S-RBD function. A total of 3014 compounds from a customized drug library were screened by molecular docking. Candidate binding and functional activity were subsequently evaluated using cellular thermal shift assays, surface plasmon resonance, immunoprecipitation, immunofluorescence, and pseudovirus-entry assays against 2019-nCoV, Delta, and Omicron pseudoviruses. DOTAP chloride (K_D_ = 49.94 μM), cefotiam hexetil hydrochloride (K_D_ = 142.26 μM), melittin (K_D_ = 34.98 μM), and teicoplanin (K_D_ = 73.58 μM) showed detectable binding to the S-RBD and inhibited Spike-mediated pseudovirus entry. Molecular docking suggested that these interactions were mediated by potential binding modes involving hydrogen bonds and π-interactions. The compounds interfered with S-RBD/hACE2 colocalisation and inhibited pseudovirus entry with variant-dependent efficacy. DOTAP chloride (25 μM) inhibited entry of the 2019-nCoV and Omicron pseudoviruses, whereas the other three compounds showed activity across the tested variants. These findings identify four compounds with RBD-binding and entry-inhibition properties for further development as entry inhibitors.

## 1. Introduction

The COVID-19 pandemic, driven by the global spread of SARS-CoV-2, remains a major public-health threat because of continuous viral evolution and the emergence of immune-evasive variants [1,2]. Despite advances in vaccines and antivirals, mutations in the viral Spike protein, particularly its receptor-binding domain (S-RBD), compromise the durability of existing therapies. The S-RBD engages human angiotensin-converting enzyme 2 (hACE2) to mediate host-cell entry [3]. Current entry-targeting strategies, including monoclonal antibodies and hACE2 decoys, have shown limited efficacy against newer lineages such as Omicron, underscoring the need for inhibitors with activity across multiple variants [4,5].

Virtual screening and molecular docking of existing compounds, coupled with biological validation, provide a practical strategy for accelerating therapeutic discovery [6,7,8]. Screening FDA-approved drugs and natural products against conserved S-RBD residues may identify agents that disrupt viral–host interactions while avoiding the lengthy timelines associated with de novo drug development [9,10,11].

Previous efforts have focused mainly on viral proteases or RNA-dependent RNA polymerase [12,13,14,15]. By contrast, direct targeting of the S-RBD, a key determinant of infectivity and immune evasion, remains underexplored. Monoclonal antibodies provide therapeutic and prophylactic benefits but are vulnerable to neutralization escape by Omicron subvariants, including BA.4 and BA.5 [16,17]. Only bebtelovimab and cilgavimab retained partial activity against these variants [18]. Their clinical use is also constrained by high production costs and the need for parenteral administration. Peptidomimetics, including the ACE2 α-helix-derived IEPFF [19], WIEPFF (W6) [19], and the computationally designed miniproteins LCB1 and LCB3 [20,21], show potent in vitro neutralization. However, their poor drug-like properties, including low oral bioavailability and metabolic instability, limit their translational potential.

Small-molecule inhibitors therefore represent a promising alternative, although this field remains in its early stages. Existing candidates include repurposed drugs such as ceftazidime [22], de novo-designed molecules such as MM3122 [23,24] and CKP-25 [25], and compounds identified through in silico screening [26,27,28,29]. These candidates often lack rigorous biological validation, show uncertain activity against emerging variants, or have incomplete preclinical profiles. Notably, MM3122 targets a conserved S-RBD–NTD junction but may lose efficacy against lineages carrying NTD mutations. Together, these limitations highlight the need for additional candidates and provide the rationale for the present study.

To address this need, we integrated in silico molecular docking with in vitro validation to systematically identify compounds that bind to the S-RBD and inhibit Spike-mediated pseudovirus entry. Among the identified compounds, DOTAP chloride, included primarily as a cationic lipid control, showed detectable RBD binding and entry inhibition, although its activity may involve non-specific mechanisms. Our screen identified lead compounds, including teicoplanin and cefotiam hexetil, that retained activity against Omicron BA.5. We further repurposed approved drugs, such as teicoplanin, as potential RBD-targeting candidates. These candidates combine established safety profiles with detectable RBD-binding properties and may serve as starting points for further development.

## 2. Results

### 2.1. Identification of 2019-nCoV S-RBD-Binding Compounds by Virtual Screening

**Figure 1:**
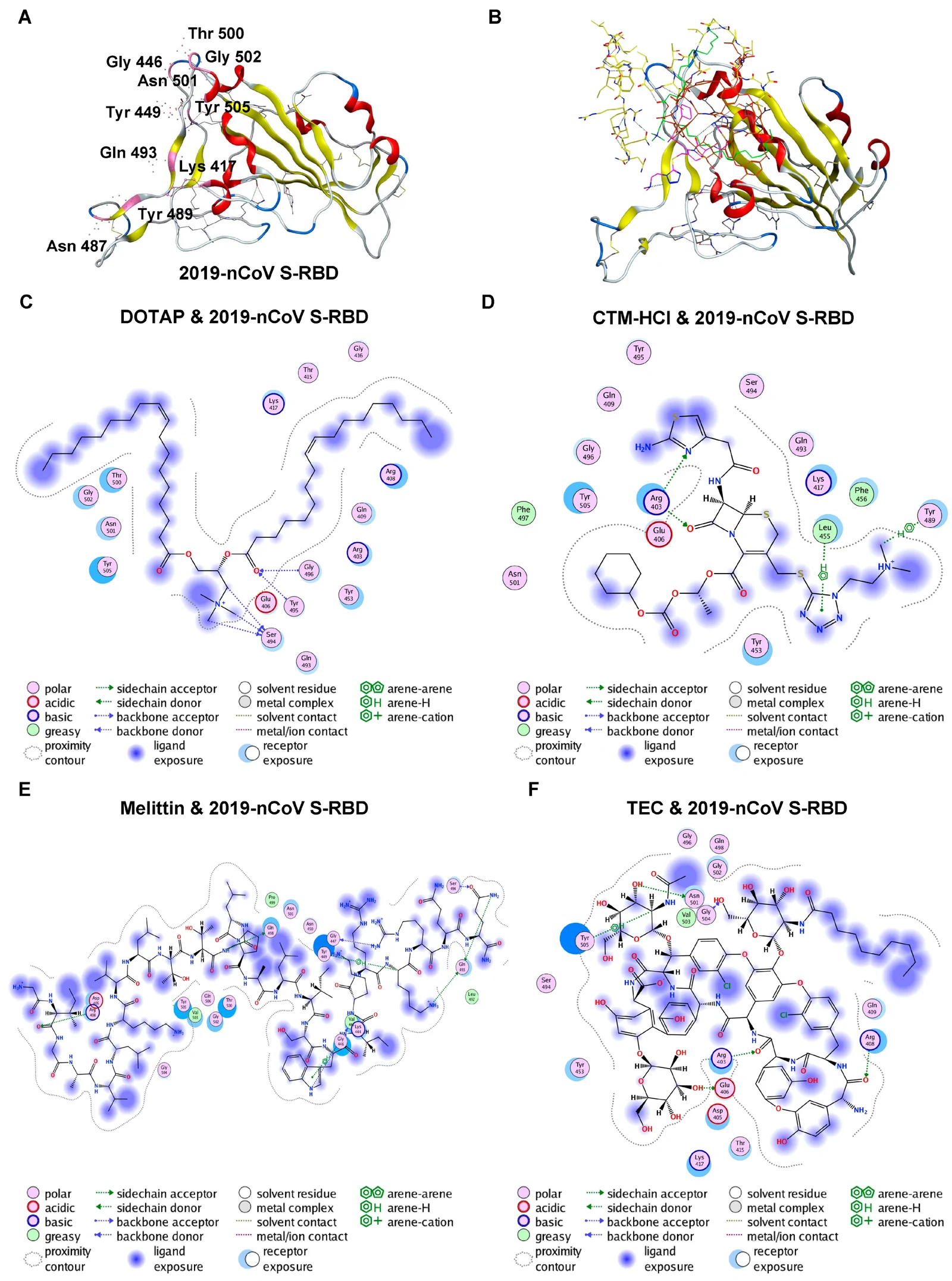
Virtual screening of 2019-nCoV S-RBD binding compounds. (**A**) Crystal structure of the 2019-nCoV S-RBD. The predicted binding hotspots of S-RBD with hACE2 are shown in pink. (**B**) Predicted binding poses of four compounds with the S-RBD. Green is DOTAP chloride (DOTAP), pink is cefotiam hexetil hydrochloride (CTM-HCl), yellow is melittin and brown is teicoplanin (TEC). Two-dimensional plots showing the interaction of (**C**) DOTAP, (**D**) CTM-HCl, (**E**) melittin and (**F**) TEC with the S-RBD are provided.

Structural analysis of the 2019-nCoV S-RBD/hACE2 complex (PDB ID: 6M0J) [30] identified ten S-RBD residues for focused analysis: Lys417, Gly446, Tyr449, Asn487, Tyr489, Gln493, Thr500, Asn501, Gly502, and Tyr505 (Figure 1A). These residues were selected because of their critical roles in viral attachment to hACE2. A structure-based virtual screening approach was subsequently applied to 3014 compounds, with these residues defined as binding hotspots. Based on the initial screening results, the 36 compounds with docking scores below −6.0 were selected for further analysis. The compound-screening workflow is shown in Figure S1. In this analysis, lower docking scores were interpreted as indicating a higher probability of binding and stronger predicted binding affinity.

Molecular docking predicted that DOTAP chloride (DOTAP, S = −7.27), cefotiam hexetil hydrochloride (CTM-HCl, S = −7.04), melittin (S = −11.40), and teicoplanin (TEC, S = −10.21) bind within the ACE2-binding pocket (Figure 1B). DOTAP formed hydrogen bonds through its quaternary ammonium cation with Ser494, while the carbon atoms of its ester bond were involved in hydrogen-bond interactions with Tyr495 and Gly496 (Figure 1C). The tetrazole ring and dimethylamino groups of CTM-HCl formed stable π–H interactions with Leu455 and Tyr489, respectively. In addition, the nitrogen atom of its thiazole ring and the carbonyl group of its β-lactam ring formed hydrogen bonds with Arg403 (Figure 1D).

Melittin exhibited a more complex interaction profile. Its peptide bond, guanidino group, and free amino group participated in hydrogen-bond interactions involving Arg408, Gln498, Ser494, Gln493, and Gly447. The carbon atoms bridging its indole group and peptide bond formed stable π–H interactions with Gly446 and Tyr449 (Figure 1E). Teicoplanin formed hydrogen bonds with Arg403, Arg408, Glu406, Asn501, and Gly504 through its amide and hydroxyl groups. The carbon atom adjacent to its acetamido group formed a stable π–H interaction with Tyr505 (Figure 1F). Bacitracin zinc (BZ) and angiotensin II (Ang II) also interacted with key residues within the S-RBD-binding site (Table S1 and Figure S2). Collectively, these in silico predictions suggested possible binding modes that may interfere with the S-RBD/hACE2 interface, which guided our subsequent experimental evaluation.

### 2.2. CETSA Assessment of S Protein–Ligand Interactions

**Figure 2:**
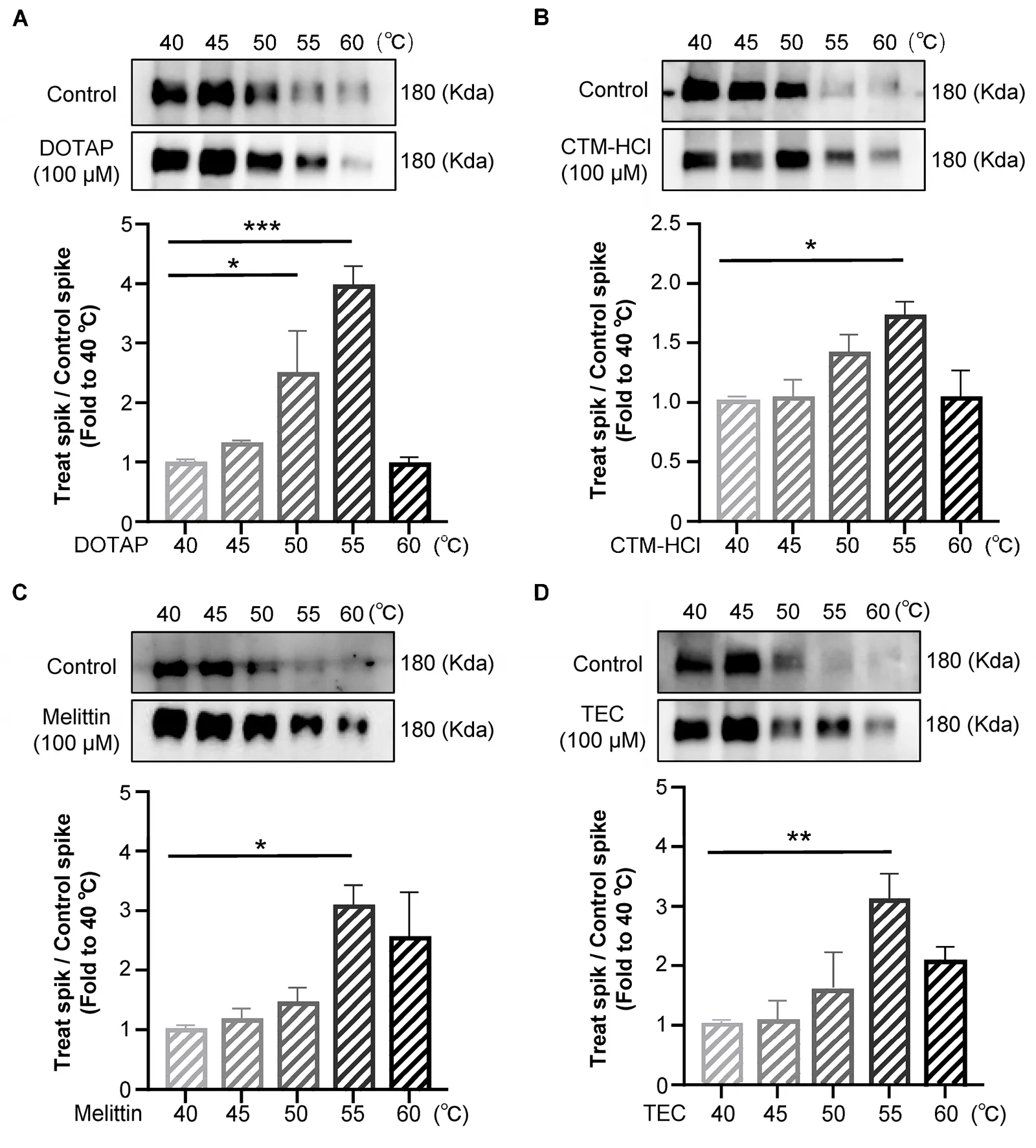
Effect of compounds on the thermal stability of 2019-nCoV S protein. CETSA analysis of the 2019-nCoV S protein was performed using HEK293T cell lysates. The cells, diluted in PBS, were subjected to freeze-thaw cycles with liquid nitrogen. The lysates were then incubated with 100 μM (**A**) DOTAP, (**B**) CTM-HCl, (**C**) Melittin or (**D**) TEC and subsequently analyzed by Western blotting. Data were collected from three independent experiments and analyzed using the one-way ANOVA, with statistical significance denoted as * *p* < 0.05, ** *p* < 0.01, and *** *p* < 0.001 compared to the 40 °C.

To experimentally evaluate the binding of the 36 top-ranked candidates to the 2019-nCoV S protein, the cellular thermal shift assay (CETSA) was performed. Lysates containing the S protein were incubated with each compound at 100 μM for 30 min, followed by thermal treatment at 55 °C for 3 min. At this single screening temperature, several compounds, including DOTAP, CTM-HCl, melittin, and teicoplanin, showed increased soluble S-protein levels compared with the vehicle-treated controls (Figure 2). Quantification of band intensities at 55 °C revealed fold-change ratios of 3.49 ± 0.50 for DOTAP, 1.64 ± 0.21 for CTM-HCl, 2.70 ± 0.52 for melittin, and 2.02 ± 0.49 for teicoplanin relative to controls (mean ± SD, *n* = 3).

The observed shifts in thermal stability provided preliminary evidence of target engagement and were consistent with the computational predictions. To more reliably assess target engagement, we performed full-curve CETSA analysis from 40 °C to 60 °C and calculated melting temperatures (Tm) for each compound (Figure S3 and Table S2). Using a positivity criterion of ΔTm ≥ 1.5 °C (based on baseline variability), 11 of the 19 evaluated compounds were identified as candidate binders for further evaluation. Among the four compounds ultimately prioritised for functional characterisation, DOTAP (ΔTm = +9.1 °C) and CTM-HCl (ΔTm = +2.2 °C) were classified as CETSA-positive, suggesting their thermal stabilisation of the S protein. In contrast, teicoplanin (ΔTm = −4.0 °C) and melittin (ΔTm = +0.7 °C) did not meet the positive threshold.

To quantitatively characterize the binding affinities of the prioritized compounds, surface plasmon resonance (SPR) assays were performed.

### 2.3. SPR Assessment of S-RBD–Compound Binding

**Figure 3:**
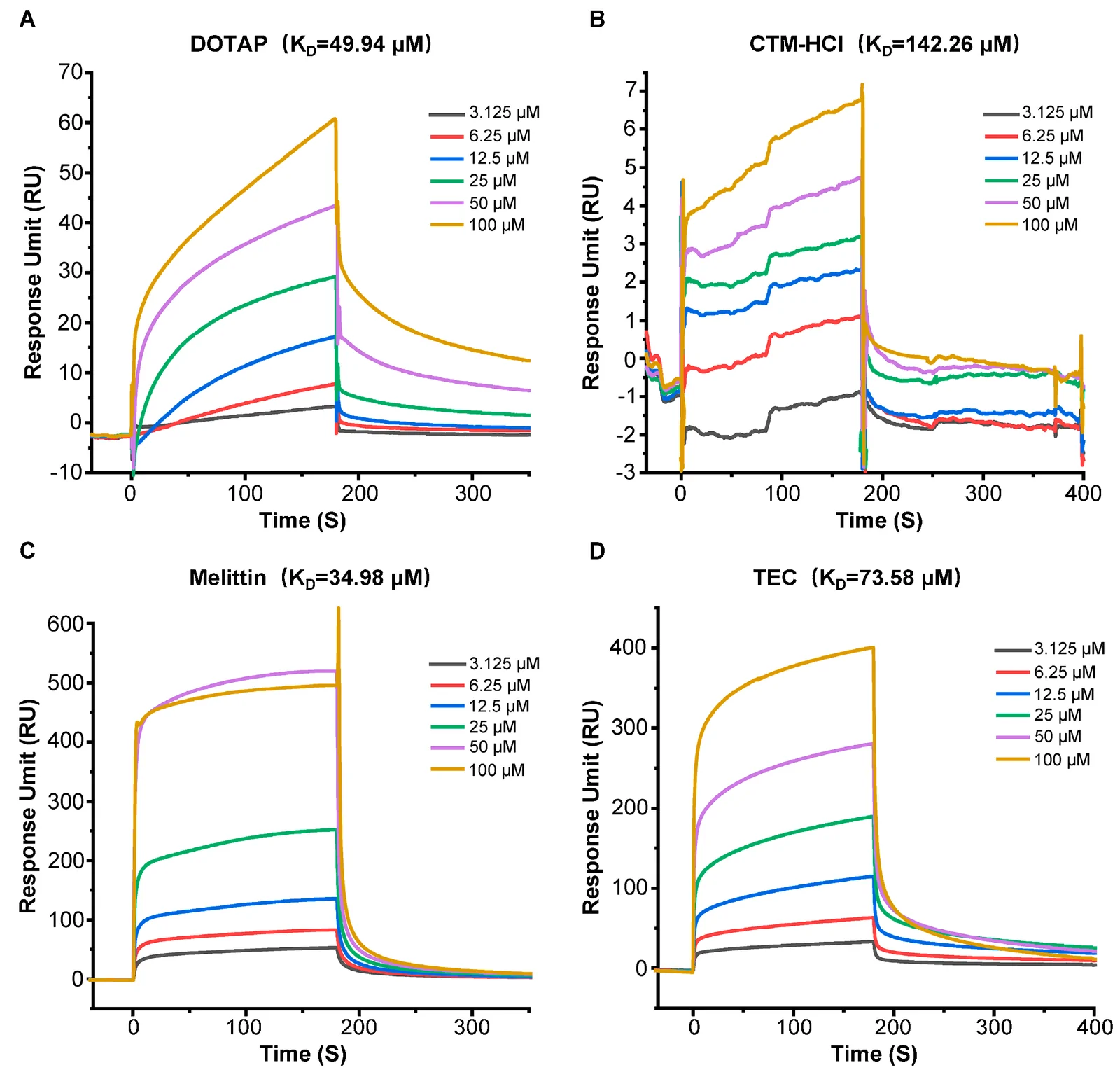
Binding affinity of compounds to 2019-nCoV S-RBD. The 2019-nCoV S-RBD was immobilized on a CM5 sensor chip using the Biacore 8K instrument. The association and dissociation curves for (**A**) DOTAP, (**B**) CTM-HCl, (**C**) Melittin and (**D**) TEC are shown. The compounds were tested at various concentrations, and the KD values were determined by fitting the data to a 1:1 binding model using Biacore Insight Evaluation Software. Fitting quality was assessed using χ^2^, Rmax, and R^2^ values (Table S3). KD represents the equilibrium dissociation constant.

To quantitatively characterise the binding interactions between the 2019-nCoV S-RBD and the prioritised compounds, SPR assays were performed. The S-RBD was covalently immobilised on a CM5 sensor chip by amine coupling, followed by the injection of serially diluted compounds at concentrations ranging from 3 to 100 μM. Real-time binding analysis revealed concentration-dependent responses for the four prioritised compounds (Figure 3), as well as for BZ and Ang II (Figure S4). The equilibrium dissociation constants (K_D_) for DOTAP, CTM-HCl, melittin, and teicoplanin were 49.94 μM, 142.26 μM, 34.98 μM, and 73.58 μM, respectively, indicating micromolar-range binding affinities. Fitting quality was evaluated using χ^2^, Rmax, and R^2^ values for the 1:1 Langmuir model; all steady-state affinity fits yielded R^2^ > 0.91, with most exceeding 0.95 (Table S3 and Figure S5). The SPR experiments included reference channel subtraction, blank injections, and solvent correction to account for non-specific binding and DMSO effects.

The observed affinity profiles were generally consistent with the thermal-stabilisation effects detected by CETSA for DOTAP and CTM-HCl, whereas teicoplanin and melittin, despite being CETSA-negative, showed detectable SPR binding. Notably, the binding responses for DOTAP, a cationic lipid, showed concentration-dependent and saturable signals, although non-specific electrostatic or membrane-like interactions cannot be fully excluded. For melittin, elevated χ^2^ and Rmax values were observed in some replicates (up to 30,658 RU^2^ and 2649 RU, respectively). In contrast, DOTAP (χ^2^ = 3.3–4.3 RU^2^, Rmax = 44–92 RU) and CTM-HCl (χ^2^ = 2.5 RU^2^, Rmax = 16 RU) consistently showed lower values across replicates. Together, these SPR measurements indicated that the four compounds are capable of binding to the S-RBD with micromolar affinities.

We next examined whether these S-RBD-binding compounds could interfere with the S protein–hACE2 interaction in cellular contexts.

### 2.4. Compounds Reduce S Protein–hACE2 Complex Formation

**Figure 4:**
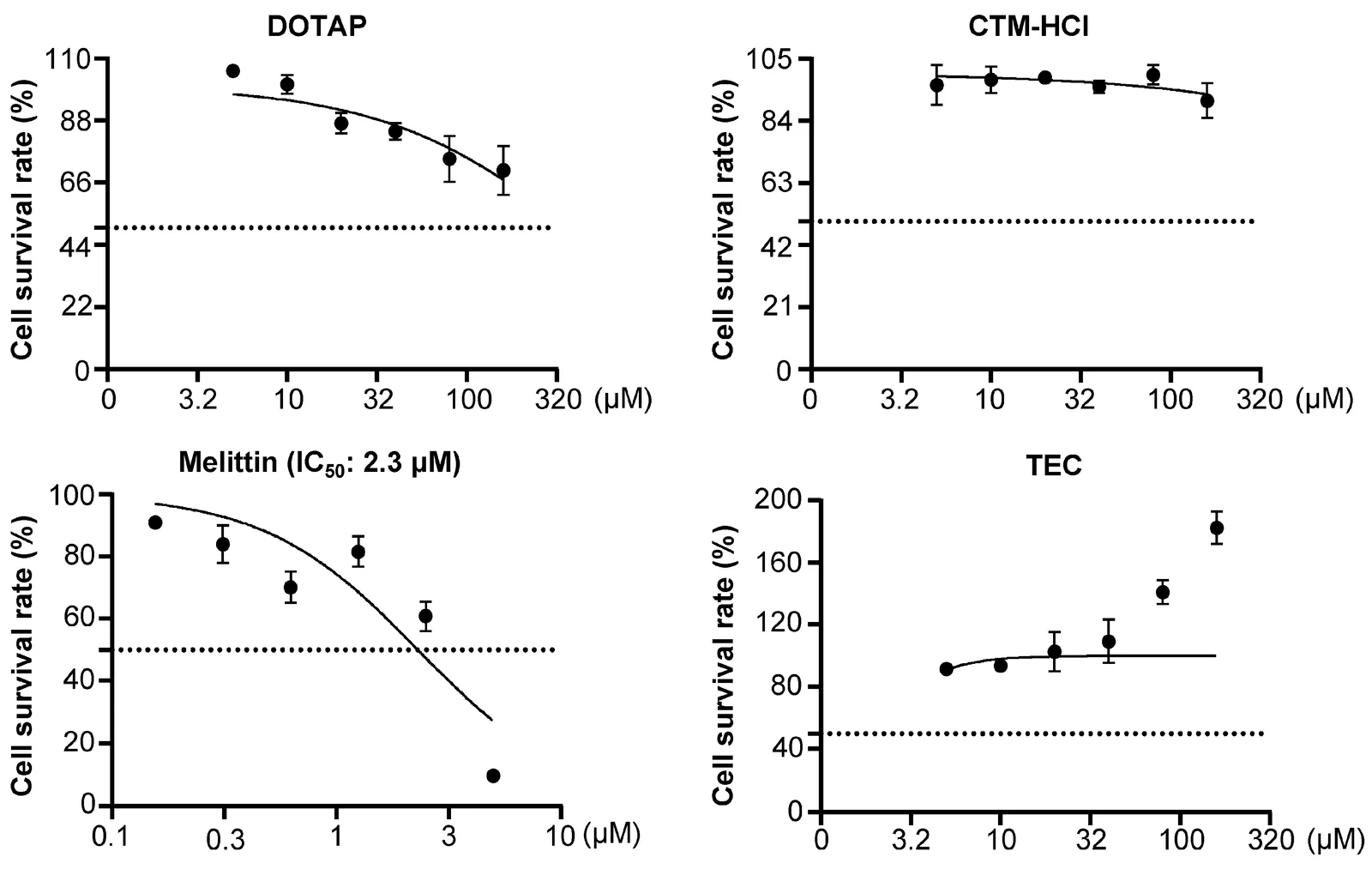
Cytotoxicity of compounds. The cytotoxicity of DOTAP, CTM-HCl, Melittin or TEC was assessed in HEK293T cells, with IC_50_ values measured at 24 h.

To evaluate whether the prioritised compounds could interfere with S protein–hACE2 interactions, MTT assays in HEK293T cells showed negligible cytotoxicity for DOTAP chloride, CTM-HCl, teicoplanin, and Ang II, with cell viability remaining above 90% after 24 h of exposure to concentrations of up to 160 μM. In contrast, melittin and BZ induced 50% cell death at concentrations of 2.3 and 79.85 μM, respectively (Figure 4 and Figure S6A).

**Figure 5:**
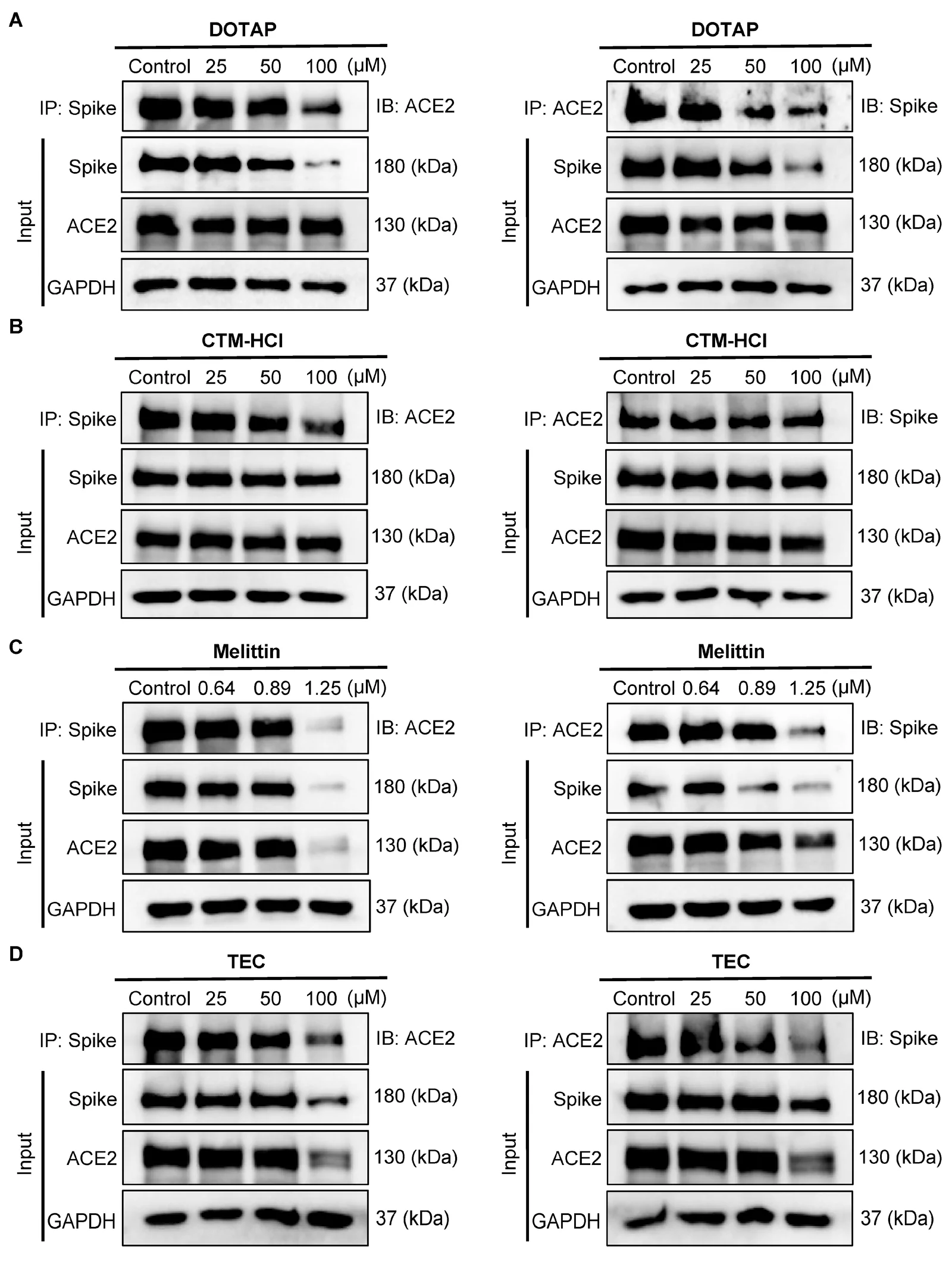
Compounds reduce complex formation of 2019-nCoV S protein with hACE2. Co-immunoprecipitation (Co-IP) assays were performed to investigate the interaction between SARS-CoV-2 Spike protein and hACE2 in HEK293T cells. Cells were treated with (**A**) DOTAP, (**B**) CTM-HCl, (**C**) Melittin or (**D**) TEC for 24 h. Immunoprecipitation was carried out using anti-hACE2 or anti-Spike antibodies, followed by immunoblotting with antibodies against hACE2, Spike, and GAPDH.

Co-immunoprecipitation assays in HEK293T cells co-transfected with S protein and hACE2 revealed distinct effects among the tested compounds. DOTAP chloride caused a dose-dependent reduction in S–hACE2 complex formation, with a 51.4% reduction in S-protein pull-downs and a 46.8% reduction in hACE2 pull-downs at 100 μM (Figure 5A). CTM-HCl produced a modest reduction in complex formation, with 32.5% and 7.9% reductions in S-protein and hACE2 pull-downs, respectively, at 100 μM (Figure 5B). Melittin also reduced co-immunoprecipitated S protein and hACE2, with 85.1% and 65.8% reductions at 1.25 μM (Figure 5C).

Teicoplanin produced a dose-dependent reduction in S–hACE2 complex formation, with a 52.2% reduction in S-protein pull-downs and a 54.4% reduction in hACE2 pull-downs at 100 μM (Figure 5D). BZ showed a similar dose-dependent effect, with a 61.8% reduction in S-protein pull-downs and a 95.7% reduction in hACE2 pull-downs at 75 μM (Figure S6B). By contrast, Ang II did not appreciably reduce S-RBD/hACE2 co-immunoprecipitation (Figure S6C).

These results indicate that DOTAP, teicoplanin, and BZ reduced S protein–hACE2 complex formation in cells, whereas CTM-HCl showed a modest effect. We next investigated whether this activity was retained against S-RBDs from major SARS-CoV-2 variants.

### 2.5. Compounds Interfere with hACE2 Binding to SARS-CoV-2 Variant S-RBDs

To evaluate whether the four lead compounds interfered with colocalisation of different SARS-CoV-2 variants, we assessed their effects on the ancestral 2019-nCoV strain and the Delta (B.1.617.2) and Omicron (BA.5) variants. Molecular docking simulations were performed using the S-RBD structures of the Delta (PDB ID: 7V8B) and Omicron (PDB ID: 7T9L) variants to predict compound–residue interactions (Figure S6D,E and Table S4).

**Figure 6:**
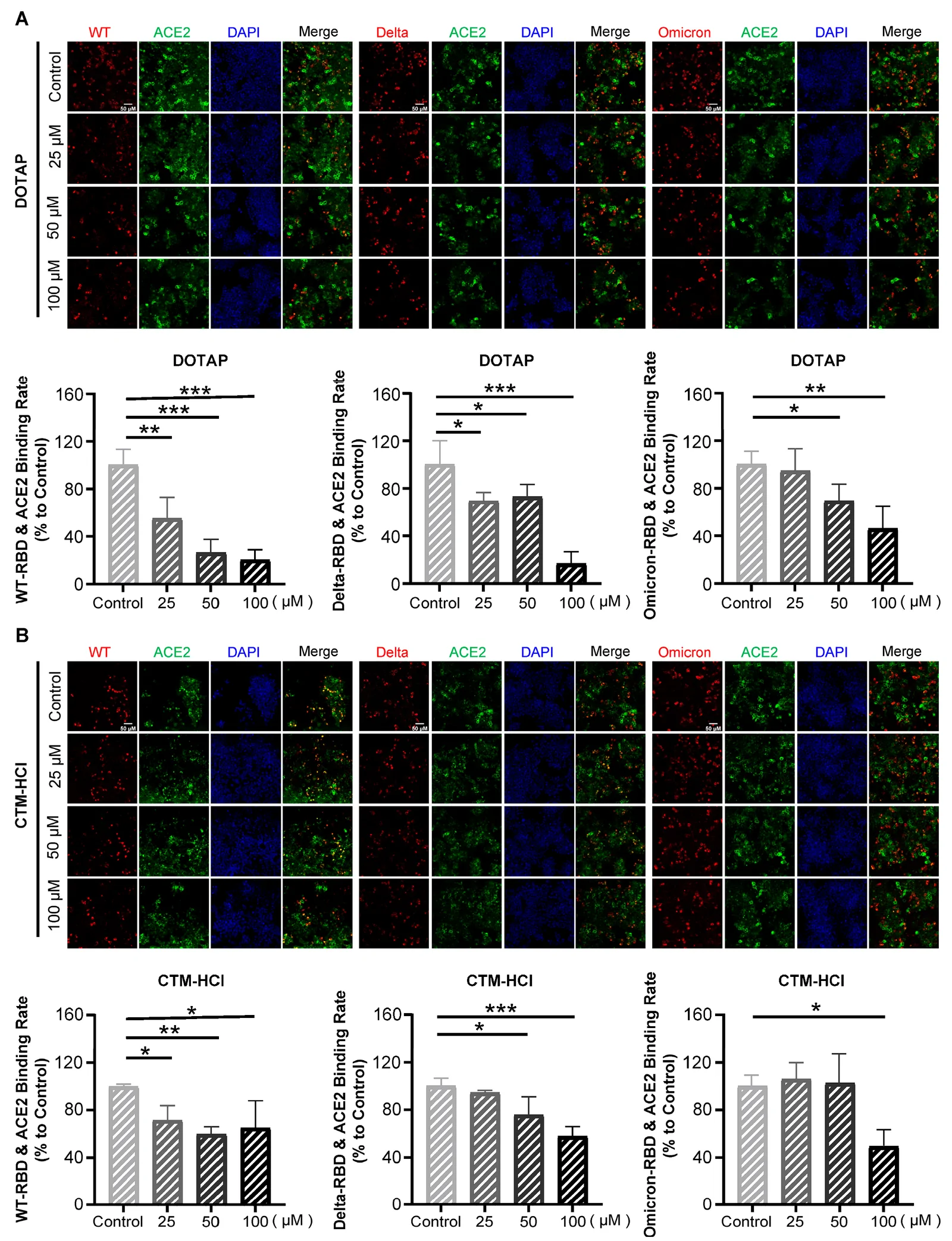

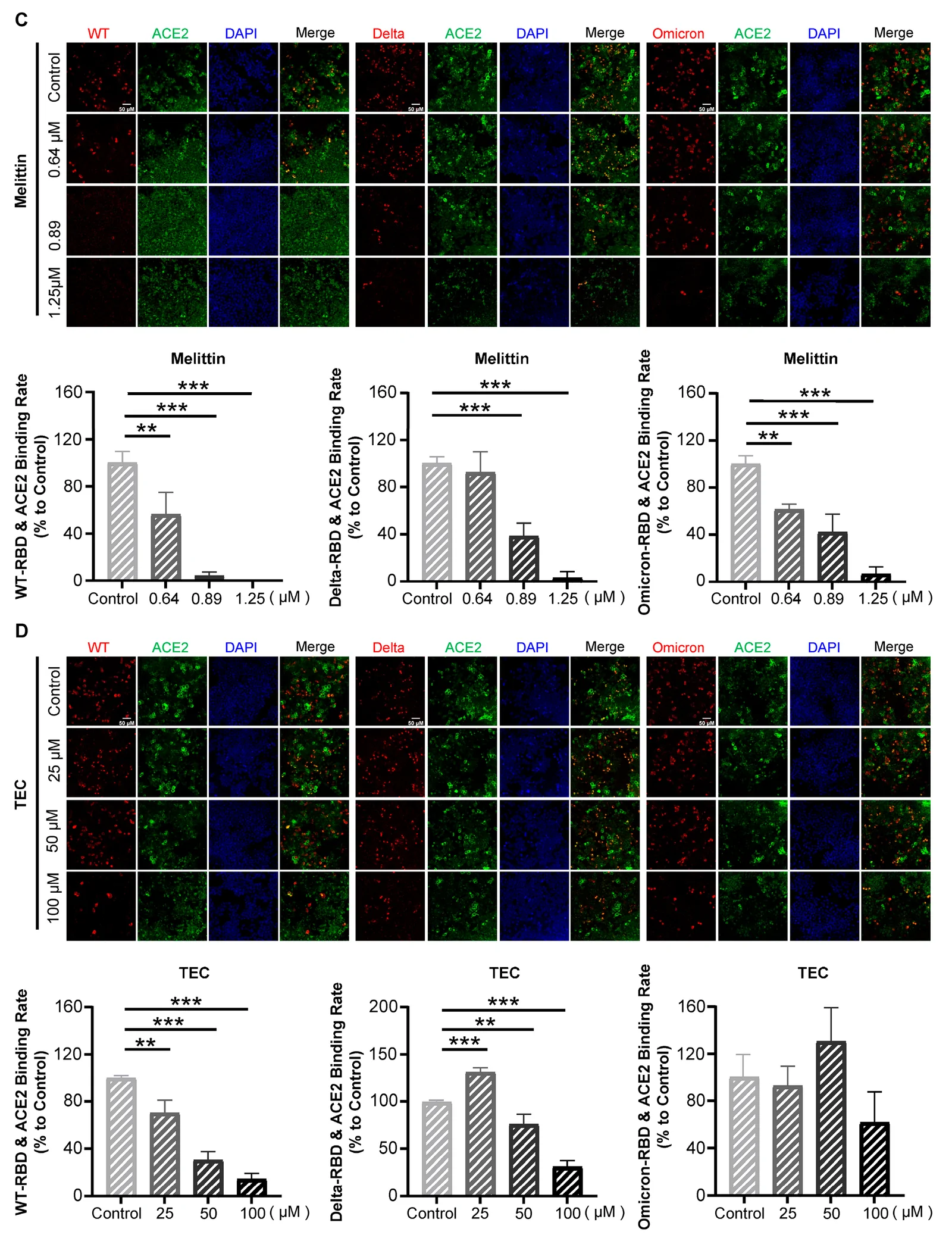
Compounds interfere with SARS-CoV-2 S protein and hACE2 co-localization. Co-localization of SARS-CoV-2 S-RBD variants (2019-nCoV, Delta, and Omicron) with hACE2 was assessed following treatment with (**A**) DOTAP, (**B**) CTM-HCl, (**C**) Melittin or (**D**) TEC in HEK293T cell for 24 h. Immunofluorescence staining was performed with red for SARS-CoV-2 S-RBD, green for hACE2, and blue for DAPI. Co-localization analysis of SARS-CoV-2 S-RBD variants and hACE2 was conducted using one-way ANOVA from three independent experiments, with statistical significance denoted as * *p* < 0.05, ** *p* < 0.01, and *** *p* < 0.001 compared to the control. Results for four compounds are shown in (**A**–**D**).

Immunofluorescence assays revealed distinct inhibition profiles across the tested variants. DOTAP chloride reduced colocalisation to all three S-RBD variants in a concentration-dependent manner (Figure 6A,C). CTM-HCl also showed similar effects against all tested strains (Figure 6B). By contrast, teicoplanin effectively reduced colocalisation of the ancestral and Delta S-RBDs but not of the Omicron S-RBD (Figure 6D).

Collectively, these results indicated that the four compounds exhibited a variant-dependent reduction in S-RBD–hACE2 colocalisation across the three tested variants (wild-type, Delta, and Omicron).

### 2.6. Compounds Reduce SARS-CoV-2 Pseudovirus Entry

To determine whether the binding and interference activities translated into inhibition of pseudovirus entry, pseudovirus-entry assays were performed using 293T/hACE2/TMPRSS2 cells. Pseudoviruses were pre-incubated with graded concentrations of each compound for 1.5 h before addition to the cells.

**Figure 7:**
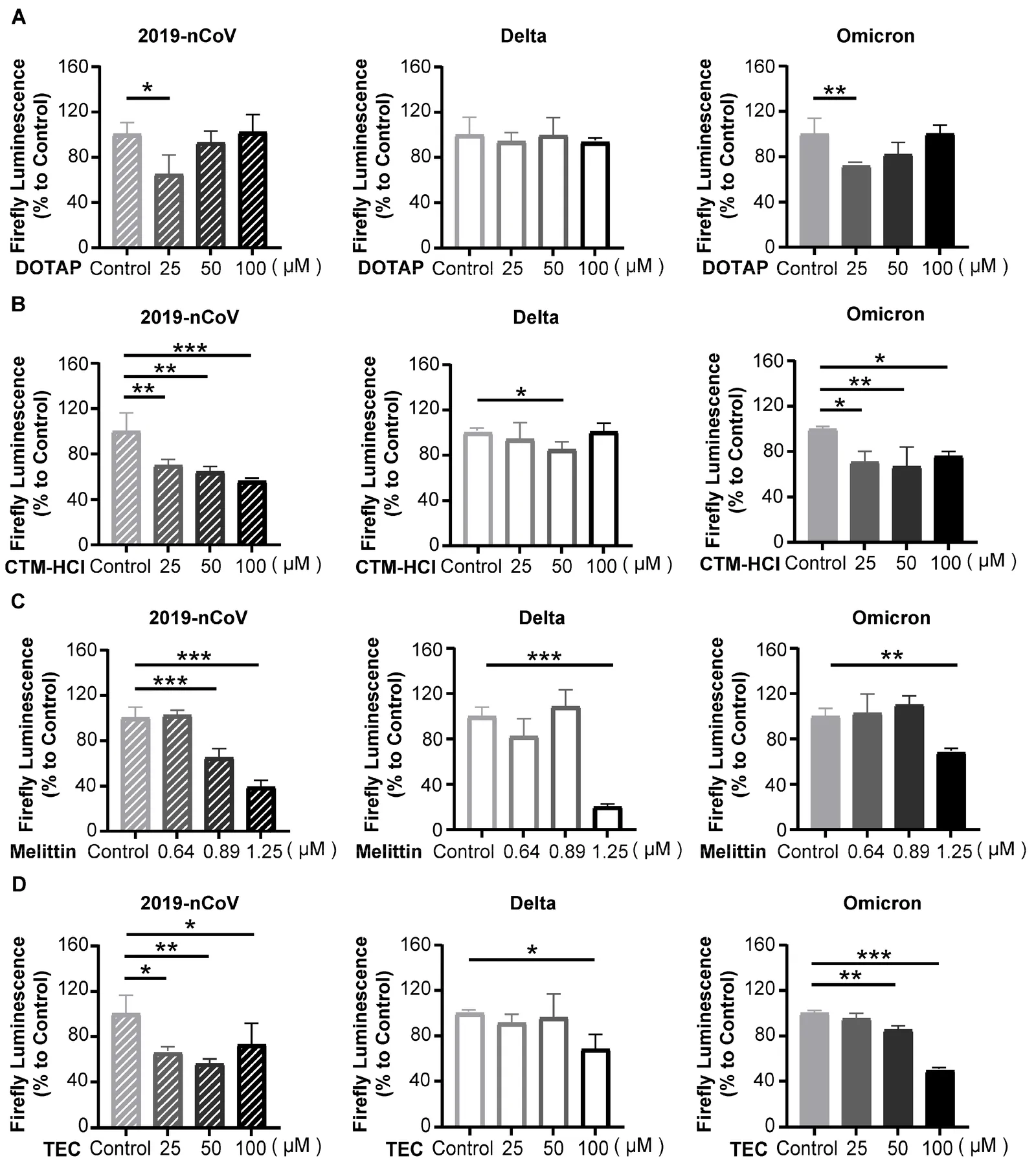
Compounds reduce SARS-CoV-2 pseudovirus entry. (**A**) DOTAP, (**B**) CTM-HCl, (**C**) Melittin or (**D**) TEC was diluted in culture medium and mixed with pseudoviruses of 2019-nCoV, Delta, and Omicron. The resulting mixtures were then added to 293T/hACE2 & TMPRSS2 cells that had been pre-seeded for infection. After 12 h of incubation, luciferase activity in the infected cells was measured to evaluate the entry inhibition of the compounds. Data were analyzed using one-way ANOVA from three independent experiments, with statistical significance indicated as * *p* < 0.05, ** *p* < 0.01, and *** *p* < 0.001 compared to the control.

Quantitative analysis revealed distinct variant-dependent inhibition profiles. DOTAP chloride at 25 μM reduced the entry of 2019-nCoV and Omicron pseudoviruses but showed limited efficacy against the Delta variant (Figure 7A). CTM-HCl reduced entry of the ancestral and Omicron strains in a concentration-dependent manner, whereas its effect on Delta entry was marginal and detectable only at 50 μM (Figure 7B). Melittin reduced entry of all three pseudoviruses at 1.25 μM (Figure 7C). Teicoplanin reduced Omicron pseudovirus entry in a concentration-dependent manner and was effective against the ancestral strain at 25–50 μM. However, its activity against Delta was observed only at 100 μM (Figure 7D).

Table S5 summarises the pseudovirus entry inhibition and preliminary selectivity indices of the four lead compounds against the three SARS-CoV-2 variants. These results indicate variant-dependent inhibition of pseudovirus entry across the three tested variants. The selectivity indices are preliminary estimates, as cytotoxicity and pseudovirus assays were performed in different cell lines (HEK293T vs. ACE2/TMPRSS2-293T).

## 3. Discussion

Based on virtual screening, we selected 36 compounds with predicted docking scores (S < −6.0) against the 2019-nCoV S-RBD. Subsequent evaluation by CETSA, SPR, and pseudovirus entry inhibition assays identified DOTAP chloride, cefotiam hexetil hydrochloride (CTM-HCl), melittin, and teicoplanin (TEC) as compounds with detectable RBD-binding and pseudovirus entry-inhibitory activity. Among these, DOTAP chloride was included primarily as a cationic lipid control; its activity may involve non-specific electrostatic or membrane-related effects, and it should not be considered a specific RBD-targeting candidate at this stage. Although teicoplanin and melittin were CETSA-negative under the conditions tested, they were retained for further evaluation based on their positive SPR-binding signals and functional activity in pseudovirus assays, consistent with the orthogonal validation strategy of our screening funnel. CETSA results can be influenced by compound solubility, membrane partitioning, or assay conditions and thus should be interpreted as preliminary evidence of target engagement rather than definitive validation.

Molecular docking simulations (Figure 1C–F) suggested that all four compounds were predicted to bind within the RBD pocket and were predicted to form π–H interactions with Gly446, Tyr449, Tyr489, and Tyr505. Although Gly496 and Arg403 are not primary hACE2-binding determinants [30], their combined contributions may stabilise the ligand–RBD complex and alter the electrostatic environment required for viral attachment. These in silico predictions were consistent with the observed SPR binding profiles (Figure 3) and pseudovirus entry inhibition data (Figure 7), suggesting a possible contribution of RBD binding to the inhibition of pseudovirus entry. However, docking-derived binding modes remain computational hypotheses and require experimental validation (e.g., site-directed mutagenesis or competition SPR).

To assess the activity against distinct variants, we further evaluated the four compounds against the Delta and Omicron variants. Immunofluorescence and pseudovirus assays showed that the compounds exhibited variant-dependent inhibition of pseudovirus entry across the three tested variants (wild-type, Delta, and Omicron), whereas DOTAP chloride exhibited weaker activity against the Delta variant. The observed variant-dependent differences in functional activity were not fully explained by the docking predictions; the current computational data do not allow definitive conclusions about the molecular basis of variant selectivity. Further structural studies are required to clarify the determinants of variant-dependent inhibition.

For CTM-HCl, the apparent discrepancy between its relatively weak SPR binding affinity (K_D_ = 142.26 μM) and its cellular activity in pseudovirus assays may reflect additional mechanisms beyond direct RBD binding, such as effects on membrane fusion, cellular uptake, or intracellular distribution. These possibilities remain speculative and were not directly tested in this study; further mechanistic investigation is required to clarify the contribution of RBD-dependent versus RBD-independent pathways to its entry-inhibitory activity.

From a translational perspective, the four compounds span a spectrum from a mechanistic tool compound to a repurposing candidate. Melittin exhibited strong in vitro entry inhibition activity. However, its very low therapeutic index (SI ~1.2) is consistent with its non-specific membrane-lytic mechanism [31,32,33] and currently precludes systemic administration. We therefore regard melittin primarily as a membrane-active peptide control for investigating viral membrane-fusion processes.

For DOTAP chloride, the SPR data showed concentration-dependent and saturable binding responses, but this does not fully exclude non-specific electrostatic or membrane-like interactions, given its cationic lipid properties. Therefore, we interpret DOTAP’s activity as potentially involving multiple non-specific mechanisms—including electrostatic interference, hydrophobic interactions, and possible membrane perturbation—rather than as evidence of specific RBD-targeted inhibition. These mechanistic possibilities are speculative and were not directly tested in this study.

DOTAP’s cationic lipid properties also interfered with standard cytotoxicity assays, preventing a reliable selectivity index calculation. Its mechanism and safety profile require further investigation using orthogonal viability assays before any translational considerations.

CTM-HCl and TEC offer important repurposing advantages [34]. Both are approved antibiotics with established safety profiles, and their therapeutic indices were favourable in the HEK293T-based cytotoxicity assays (preliminary estimates). Their known adverse effects, including gastrointestinal toxicity associated with CTM-HCl and nephrotoxicity and ototoxicity associated with teicoplanin, are primarily linked to long-term, high-dose systemic administration [35]. Repurposing these compounds as short-term, locally administered agents through topical or local delivery routes could preserve their entry-inhibitory activity while minimising systemic toxicity. The long half-life and favourable tissue distribution of teicoplanin [36] further support its potential as a long-acting local prophylactic. However, such applications require formulation, pharmacokinetic, pulmonary delivery, and animal efficacy studies, which are beyond the scope of this work and should be considered as future research directions.

Several limitations of this study should be acknowledged. First, the functional experiments were performed using pseudoviruses rather than authentic SARS-CoV-2; thus, our conclusions are confined to inhibition of Spike-mediated pseudovirus entry and cannot be extrapolated to complete viral replication or live-virus infection without further validation. Second, the cytotoxicity and pseudovirus assays were performed in different cell lines (HEK293T vs. ACE2/TMPRSS2-293T), so the selectivity indices are preliminary estimates and require confirmation in the same cell system. Third, only three variants (wild-type, Delta, and Omicron) were evaluated; the term “broad-spectrum” is therefore not applicable. Fourth, the binding affinities are in the micromolar range, which are relatively weak for protein–protein interaction inhibitors. Fifth, the docking-derived binding modes are computational predictions and have not been experimentally validated (e.g., by mutagenesis or competition SPR). Sixth, CETSA was performed at a single compound concentration and should be considered preliminary evidence of target engagement rather than definitive validation. Seventh, DOTAP’s mechanism may involve non-specific effects and requires orthogonal validation. Eighth, melittin’s narrow therapeutic window precludes its development as a therapeutic candidate. Ninth, no in vivo validation was performed. These limitations provide clear directions for future studies, including authentic virus testing, respiratory epithelial cell models, and affinity optimisation of the identified leads.

In summary, we identified diverse S-RBD-targeting compounds through an integrated computational and experimental approach and systematically evaluated their binding profiles, variant activity, and translational potential. Teicoplanin and CTM-HCl, as approved drugs with established safety profiles, may offer repurposing advantages; however, given their micromolar affinities and the lack of authentic virus and in vivo data, they should be considered as preliminary leads requiring substantial further optimisation and validation. DOTAP chloride warrants further investigation primarily as a mechanistic probe or cationic lipid control, with orthogonal assays needed to clarify its specificity. Melittin remains valuable as a membrane-active peptide control for in vitro studies. This tiered strategy provides candidate molecules and a research framework for further studies against evolving coronaviruses.

## 4. Materials and Methods

### 4.1. Compounds

A customised drug library containing 3014 compounds was purchased from Selleck Chemicals (Houston, TX, USA). Of these compounds, 57.6% were FDA-approved drugs and 42.4% were natural products. All compounds were analyzed by high-performance liquid chromatography (HPLC) and exhibited a purity of >95%.

### 4.2. Virtual Screening

Molecular docking was performed using MOE software (version 2019.01; Chemical Computing Group, Montreal, QC, Canada). The compounds were energy-minimised using the MMFF94x force field implemented in MOE. The crystal structures of the 2019-nCoV S-RBD (PDB ID: 6M0J), Delta S-RBD (PDB ID: 7V8B), and Omicron S-RBD (PDB ID: 7T9L) were retrieved from the Protein Data Bank (PDB; https://www.rcsb.org/, accessed on 20 March 2022).

All structures were preprocessed using the Structure Preparation module in MOE. This procedure included the addition of hydrogen atoms, optimization of protonation states, and local energy minimisation. For docking, the S-RBD was defined as the receptor. The binding sites were specified according to key interface residues identified from the RBD–hACE2 complex structure and previous reports: 2019-nCoV S-RBD, Lys417, Gly446, Tyr449, Asn487, Tyr489, Gln493, Thr500, Asn501, Gly502, and Tyr505 [30,37]; Delta S-RBD, Lys417, Gly446, Tyr449, Ala475, Asn487, Tyr489, Gln493, Gly496, Gln498, Thr500, and Gly502 [38,39]; and Omicron S-RBD, Tyr453, Asn477, Arg493, Ser496, Arg498, Thr500, Tyr501, Gly502, and His505 [38,40].

A semi-flexible docking protocol was used, with the receptor kept rigid and the ligands fully flexible. The binding site was defined within a 10 Å radius of the predicted binding pocket. The London dG scoring function was applied for initial pose evaluation, and force-field-based refinement with the GBVI/WSA dG scoring function for final ranking. Final compound prioritisation was based on the docking score (S-score < −6.0), combined with visual inspection of hydrogen-bond and electrostatic interactions with the key interface residues.

### 4.3. Cells, Plasmids, and Pseudoviruses

HEK293T cells were purchased from the American Type Culture Collection (ATCC), and the 293T/hACE2/TMPRSS2 stable cell line was purchased from Sino Biological (Beijing, China). Cells were maintained in Dulbecco’s modified Eagle’s medium (DMEM; SH30022.01, HyClone, Logan, UT, USA) supplemented with 10% foetal bovine serum (FBS; 10999-141, Gibco, Grand Island, NY, USA) and 1% penicillin–streptomycin at 37 °C in 5% CO_2_. Pseudoviruses bearing the Spike proteins of 2019-nCoV (PSV001), B.1.617.2 (PSV011), and Omicron BA.5 (PSV023) were obtained from Sino Biological.

Plasmids encoding the Spike RBDs of 2019-nCoV (VG40592-CF), B.1.617.2 (VG40864-UT), and BA.4/BA.5/BA.5.2 (VG40908-CH), as well as the pCDNA3.1(+)-2019-nCoV-Spike (P17428) and pEGFP-N1-hACE2 (P19644) plasmids, were purchased from Sino Biological and Miaoling Biology (Beijing, China), respectively. His-tagged 2019-nCoV S-RBD protein was obtained from the Research Dog Instrument Testing Platform (Wuhan, China).

Additional reagents included methyl thiazolyl diphenyl tetrazolium bromide (MTT, Beyotime, Shanghai, China), 4% paraformaldehyde, Triton X-100, DAPI (Beyotime, Shanghai, China), bovine serum albumin (BSA, Sigma-Aldrich, St. Louis, MO, USA), the BCA protein assay kit (Thermo Fisher Scientific, Waltham, MA, USA), the PageRuler™ prestained protein ladder (Thermo Fisher Scientific), and a hypersensitive ECL Western HRP substrate (Zen BioScience, Chengdu, China).

### 4.4. Cellular Thermal Shift Assay

Cell suspensions in phosphate-buffered saline (PBS) were subjected to three freeze–thaw cycles in liquid nitrogen. The suspensions were then centrifuged at 13,500 rpm for 20 min at 4 °C. The soluble fraction containing the S protein was collected as the lysate.

The lysates were diluted with PBS and divided into aliquots. One aliquot was treated with solvent as the control, whereas the remaining aliquots were treated with each compound at 100 μM. After incubation at room temperature for 30 min, the lysates were heated for 3 min at 40, 45, 50, 55, or 60 °C.

The samples were then cooled at room temperature for 3 min and centrifuged at 13,500 rpm for 20 min at 4 °C. The supernatants were mixed with 5× loading buffer and denatured at 100 °C for 10 min. Equal volumes of the denatured lysates were separated by SDS-PAGE and analysed by Western blotting.

For the four prioritised compounds, experiments were performed with three independent biological replicates, and data are presented as mean ± SD. For the remaining compounds, single-experiment values from the primary screening are reported.

Band intensities were quantified using ImageJ software 1.54s13 and normalised to the 40 °C signal for each replicate. Tm values were determined by linear interpolation at half-maximal signal (the midpoint between the maximum and minimum signals) from the normalised melting curves. ΔTm was calculated as Tm (compound) − Tm (control). Compounds with ΔTm ≥ 1.5 °C were considered positive.

### 4.5. Surface Plasmon Resonance Assay

SPR binding assays were performed using a Biacore 8K instrument (Cytiva, Marlborough, MA, USA). The 2019-nCoV S-RBD protein was immobilised on a CM5 sensor chip by amine coupling in 10 mM sodium acetate buffer (pH 5.0). The final protein concentration was 60 μg/mL, and the immobilisation level was approximately 5000 response units (RU).

The running buffer consisted of PBS (pH 7.4) containing 5% DMSO and 0.5% Tween 20. For binding measurements, the analytes were injected at serially diluted concentrations (3–100 μM) in the running buffer at a flow rate of 30 μL/min. Each interaction was monitored using a contact time of 180 s and a dissociation time of 180 s.

Data processing included reference channel subtraction, blank injections, and solvent correction to account for non-specific binding and DMSO effects. The multi-concentration sensorgrams were globally fitted using a 1:1 Langmuir binding model with Biacore evaluation software v3.0. The association rate constant (ka) and dissociation rate constant (kd) were determined simultaneously, and the equilibrium dissociation constant (K_D_) was calculated as K_D_ = kd/ka. Fitting quality was assessed using R^2^ values, χ^2^, and Rmax parameters. The complete fitting parameters for each replicate are provided in Table S3.

Three independent experiments were performed for each compound, each with a freshly prepared concentration series. Data were plotted using Origin 2021 software. Representative sensorgrams are shown; sensorgrams were not overlaid due to inter-batch variability in the measured K_D_ values. The relatively high immobilisation level (~5000 RU) is acknowledged as a potential limitation, as it may contribute to mass-transfer or rebinding artifacts.

### 4.6. Cell Viability Assay

HEK293T cells were seeded in 96-well plates at a density of 8 × 10^4^ cells per well and allowed to adhere overnight. The following day, the cells were treated with graded concentrations of the compounds. After 24 h of incubation, the MTT solution was added to the culture medium at a final concentration of 0.5 mg/mL. The plates were then incubated for an additional 4 h at 37 °C.

The resulting formazan crystals were dissolved in 100 μL of DMSO. Absorbance was measured at 490 nm using a Synergy Neo2 multifunctional microplate reader (Agilent, Santa Clara, CA, USA).

### 4.7. Western Blot Analysis

HEK293T or 293T/hACE2/TMPRSS2 cells were lysed in RIPA buffer (P0013C, Beyotime) containing 50 mM Tris-HCl (pH 7.4), 150 mM NaCl, 1% NP-40, 0.5% sodium deoxycholate, 0.1% SDS, and a protease inhibitor cocktail. Protein concentrations were determined using a BCA assay.

The lysates were mixed with loading buffer and denatured at 100 °C for 10 min. Equal amounts of protein were separated by 8–10% SDS-PAGE and transferred onto PVDF membranes (ISEQ00010, Millipore, Billerica, MA, USA). The membranes were incubated with primary antibodies at a dilution of 1:1000, followed by species-specific HRP-conjugated secondary antibodies at a dilution of 1:4000. Immunoreactive bands were visualized using an ECL detection kit. GAPDH was used as the loading control. Band intensities were quantified using ImageJ software (National Institutes of Health, Bethesda, MD, USA).

The primary antibodies were the anti-SARS-CoV-2 Spike antibody (GTX632604, GeneTex, Southern California, CA, USA), the anti-hACE2 antibody (21115-1-AP, Proteintech, Chicago, IL, USA), and the anti-GAPDH antibody (2118S, Cell Signaling Technology, Danvers, MA, USA). The secondary antibodies were goat anti-rabbit IgG H&L (511203) and goat anti-mouse IgG H&L (511103), both obtained from Zen BioScience.

### 4.8. Co-Immunoprecipitation Assay

HEK293T cells were cultured in 6 cm dishes and co-transfected with plasmids encoding the 2019-nCoV Spike protein and pEGFP-N1-hACE2 using polyethyleneimine (PEI; 9002-98-6, Sigma-Aldrich). After 4 h of serum-free incubation, the medium was replaced with fresh culture medium containing graded concentrations of the compounds. The cells were then incubated for an additional 24 h.

The cells were washed with cold PBS and lysed using immunoprecipitation lysis buffer (G2038, Servicebio, Wuhan, China) supplemented with phenylmethylsulphonyl fluoride (PMSF; BL507A, Biosharp, Beijing, China). After centrifugation at 13,500 rpm for 10 min at 4 °C, 70 μL of the lysate was mixed with loading buffer and denatured at 100 °C for 10 min for input analysis. Western blotting was performed according to the procedure described above.

For immunoprecipitation, 320 μL of the supernatant was incubated overnight at 4 °C with either an anti-hACE2 antibody or an anti-Spike antibody. The following day, 40 μL of Protein A/G PLUS-Agarose (sc-2003, Santa Cruz Biotechnology, Santa Cruz, CA, USA) was added, and the mixture was incubated at 4 °C for 4 h. The immunoprecipitants were washed four times and boiled in 2× SDS loading buffer at 100 °C for 10 min. The samples were centrifuged at 11,000 rpm for 1 min at 4 °C, and the supernatants were collected for Western blot analysis.

### 4.9. Cellular Immunofluorescence Analysis

HEK293T cells were plated in 15 mm glass-bottom culture dishes at a density of 3 × 10^4^ cells per dish. The cells were allowed to adhere overnight at 37 °C in a humidified incubator containing 5% CO_2_.

Plasmids encoding the S-RBD variants of 2019-nCoV, B.1.617.2, and BA.4/BA.5/BA.5.2, together with pEGFP-N1-hACE2, were co-transfected into the cells using PEI. After 4 h of serum-free incubation, the medium was replaced with fresh culture medium containing graded concentrations of the compounds. The cells were then incubated for an additional 24 h.

The cells were fixed with 4% paraformaldehyde for 20 min and permeabilised with 0.1% Triton X-100 (ST1723, Beyotime, Shanghai, China) for 8 min at room temperature. After blocking with 5% BSA for 30 min, the cells were incubated overnight at 4 °C with an anti-SARS-CoV-2 S-RBD antibody (1:200; 40592-MM117, Sino Biological, Beijing, China). An Alexa Fluor 594-conjugated secondary antibody (1:500; 550042, Zen BioScience) was used for detection. Nuclei were stained with DAPI (1 μg/mL) for 15 min.

Images were acquired using a Leica TCS SP8 confocal microscope (Leica Microsystems, Wetzlar, Germany). The percentage of cells exhibiting colocalized S-RBD and hACE2 signals was calculated relative to the total number of cells in each treatment group using ImageJ software. The values were normalised to those of the control group to determine the relative colocalisation rate. Data were obtained from three independent experiments and analysed using one-way ANOVA followed by Dunnett’s post-hoc test for multiple comparisons.

### 4.10. Pseudovirus Entry Assay

293T/hACE2/TMPRSS2 stable cells were seeded in 96-well plates at a density of 4 × 10^4^ cells per well. Pseudoviruses bearing the Spike proteins of 2019-nCoV, B.1.617.2, or Omicron BA.5 (MOI = 0.5) were incubated with serially diluted compounds at 37 °C for 1.5 h. The mixtures were then added to the cells and incubated at 37 °C for 12 h to allow viral entry.

After incubation, the cells were washed with PBS to remove unbound virus and cultured further at 37 °C. At 24 h post-infection, the cells were lysed, and 20 μL of each lysate was mixed with 100 μL of the Luciferase Assay Reagent (Promega, Madison, WI, USA). Luminescence was measured using a multifunctional microplate reader (BioTek, Winooski, VT, USA). Cell-only and virus-only controls were included in each assay as negative and positive infection controls, respectively.

The entry inhibition rate was calculated as

$$Inhibition\left(\%\right)=\left[1-\frac{Test\;well\;signal-Cell\text{-}only\;control\;signal}{Virus\text{-}only\;control\;signal-Cell\text{-}only\;control\;signal}\right]\times 100\%.$$

### 4.11. Statistical Analysis

Data are presented as the mean ± SD from at least three independent experiments. Statistical analyses were performed using GraphPad Prism 9. Statistical comparisons among multiple groups were performed using one-way analysis of variance (ANOVA). The significance thresholds were defined as follows: * *p* < 0.05, ** *p* < 0.01, and *** *p* < 0.001.

## Data Availability

The raw data supporting the conclusions of this article are openly available in the Zenodo repository at https://doi.org/10.5281/zenodo.21641801.

## Supplementary Materials

The following supporting information can be downloaded at https://www.mdpi.com/article/10.3390/ijms27188136/s1.

## Abbreviations

The following abbreviations are used in this manuscript:

BSA	Bovine serum albumin
COVID-19	Coronavirus disease 2019
CETSA	Cellular thermal shift assay
CO-IP	Co-immunoprecipitation
DMSO	Dimethyl sulfoxide
ECL	Enhanced chemiluminescence
FBS	Fetal bovine serum
hACE2	Human angiotensin-converting enzyme 2
MTT	Methylthiazolyldiphenyl-tetrazolium bromide
PDB	Protein data bank
PEI	Polyethylenimine
PBS	Phosphate-buffered saline
q-PCR	Real-time quantitative polymerase chain reaction
RBD	Receptor-binding domain
RBM	Receptor-binding motif
SARS-CoV-2	Severe acute respiratory syndrome coronavirus 2
SDS-PAGE	Sodium dodecyl sulfate–polyacrylamide gel electrophoresis
SPR	Surface plasmon resonance
Teicoplanin	TEC
TMPRSS2	Transmembrane protease serine 2

## References

[1] Zhu N., Zhang D., Wang W., Li X., Yang B., Song J., Zhao X., Huang B., Shi W., Lu R. (2020). A Novel Coronavirus from Patients with Pneumonia in China, 2019. N. Engl. J. Med..

[2] Hirabara S.M., Serdan T.D.A., Gorjao R., Masi L.N., Pithon-Curi T.C., Covas D.T., Curi R., Durigon E.L. (2022). SARS-COV-2 Variants: Differences and Potential of Immune Evasion. Front. Cell. Infect. Microbiol..

[3] Tregoning J.S., Flight K.E., Higham S.L., Wang Z., Pierce B.F. (2021). Progress of the COVID-19 vaccine effort: Viruses, vaccines and variants versus efficacy, effectiveness and escape. Nat. Rev. Immunol..

[4] Rebelo M., Tang C., Coelho A.R., Labão-Almeida C., Schneider M.M., Tatalick L., Ruivo P., de Miranda M.P., Gomes A., Carvalho T. (2023). De Novo Human Angiotensin-Converting Enzyme 2 Decoy NL-CVX1 Protects Mice From Severe Disease After Severe Acute Respiratory Syndrome Coronavirus 2 Infection. J. Infect. Dis..

[5] Baum A., Fulton B.O., Wloga E., Copin R., Pascal K.E., Russo V., Giordano S., Lanza K., Negron N., Ni M. (2020). Antibody cocktail to SARS-CoV-2 spike protein prevents rapid mutational escape seen with individual antibodies. Science.

[6] Abosheasha M.A., El-Gowily A.H., Elfiky A.A. (2021). Potential antiviral properties of antiplatelet agents against SARS-CoV-2 infection: An in silico perspective. J. Thromb. Thrombolysis.

[7] Berhe H.G., Birhan Y.S., Beshay B.Y., Habib H.J., Hymete A., Bekhit A.A. (2024). Synthesis, antileishmanial, antimalarial evaluation and molecular docking study of some hydrazine-coupled pyrazole derivatives. BMC Chem..

[8] Chen L., Chen S., Li P., Zhao X., Sun P., Liu X., Wei H., Jiang X., Zhan Z., Wang J. (2024). Exploration of the mechanism of Qinglongyi-Buguzhi drug pair in treating vitiligo based on network pharmacology, molecular docking and experimental verification. J. Ethnopharmacol..

[9] Awad I.E., Abu-Saleh A.A.-A.A., Sharma S., Yadav A., Poirier R.A. (2020). High-throughput virtual screening of drug databanks for potential inhibitors of SARS-CoV-2 spike glycoprotein. J. Biomol. Struct. Dyn..

[10] Almatarneh M.H., Alqaisi A.M., Al-Shanti A.N., Abu-Saleh A.A.-A.A., Al-Sheraideh M.S., Zhao Y., Uddin K.M. (2025). Computational Repurposing of Relatively Large Drugs for the Receptor Binding Domain of SARS-CoV-2 Spike Protein. ChemistrySelect.

[11] de Oliveira O.V., Rocha G.B., Paluch A.S., Costa L.T. (2020). Repurposing approved drugs as inhibitors of SARS-CoV-2 S-protein from molecular modeling and virtual screening. J. Biomol. Struct. Dyn..

[12] Vuong W., Khan M.B., Fischer C., Arutyunova E., Lamer T., Shields J., Saffran H.A., McKay R.T., van Belkum M.J., Joyce M.A. (2020). Feline coronavirus drug inhibits themain protease of SARS-CoV-2 andblocks virus replication. Nat. Commun..

[13] Quan B.-X., Shuai H., Xia A.-J., Hou Y., Zeng R., Liu X.-L., Lin G.-F., Qiao J.-X., Li W.-P., Wang F.-L. (2022). An orally available Mpro inhibitor is effective against wild-type SARS-CoV-2 and variants including Omicron. Nat. Microbiol..

[14] Jayk Bernal A., Gomes da Silva M.M., Musungaie D.B., Kovalchuk E., Gonzalez A., Delos Reyes V., Martín-Quirós A., Caraco Y., Williams-Diaz A., Brown M.L. (2022). Molnupiravir for Oral Treatment of Covid-19 in Nonhospitalized Patients. N. Engl. J. Med..

[15] Paladugu S., Donato A.A. (2020). Recommendations for caring for patients with severe and nonsevere COVID-19. Ann. Intern. Med..

[16] Bruel T., Stéfic K., Nguyen Y., Toniutti D., Staropoli I., Porrot F., Guivel-Benhassine F., Bolland W.-H., Planas D., Hadjadj J. (2022). Longitudinal analysis of serum neutralization of SARS-CoV-2 Omicron BA.2, BA.4, and BA.5 in patients receiving monoclonal antibodies. Cell Rep. Med..

[17] Yamasoba D., Kosugi Y., Kimura I., Fujita S., Uriu K., Ito J., Sato K. (2022). Neutralisation sensitivity of SARS-CoV-2 omicron subvariants to therapeutic monoclonal antibodies. Lancet Infect. Dis..

[18] Takashita E., Yamayoshi S., Simon V., van Bakel H., Sordillo E.M., Pekosz A., Fukushi S., Suzuki T., Maeda K., Halfmann P. (2022). Efficacy of Antibodies and Antiviral Drugs against Omicron BA.2.12.1, BA.4, and BA.5 Subvariants. N. Engl. J. Med..

[19] Liu Z., Wei Y., Zhang M., Zhu X., Liu K. (2024). Development of Novel Peptide Inhibitors Adapted to the Surface Property and Morphology of S Protein RBD. Int. J. Pept. Res. Ther..

[20] Behbahanipour M., Navarro S., Bárcenas O., Garcia-Pardo J., Ventura S. (2024). Bioengineered self-assembled nanofibrils for high-affinity SARS-CoV-2 capture and neutralization. J. Colloid Interface Sci..

[21] Fornt-Suñé M., Puertas M.C., Martinez-Picado J., García-Pardo J., Ventura S. (2024). Protein Nanoparticles for Targeted SARS-CoV-2 Trapping and Neutralization. Adv. Healthc. Mater..

[22] Zheng Y.-Z., Liu Z.-Y., Li Y., Lv X.-Y., Wu Y., Huang M.-W., Pan X.-C., Chen J.-F., Lin C.-D. (2023). Ceftazidime exhibits a broad inhibition to the infection of SARS-CoV-2 prototype and Omicron variant in vitro by blocking spike protein-ACE2 interaction. Acta Pharmacol. Sin..

[23] Boon A.C.M., Bricker T.L., Fritch E.J., Leist S.R., Gully K., Baric R.S., Graham R.L., Troan B.V., Mahoney M., Janetka J.W. (2024). Efficacy of host cell serine protease inhibitor MM3122 against SARS-CoV-2 for treatment and prevention of COVID-19. J. Virol..

[24] Mahoney M., Damalanka V.C., Tartell M.A., Chung D.H., Lourenço A.L., Pwee D., Mayer Bridwell A.E., Hoffmann M., Voss J., Karmakar P. (2021). A novel class of TMPRSS2 inhibitors potently block SARS-CoV-2 and MERS-CoV viral entry and protect human epithelial lung cells. Proc. Natl. Acad. Sci. USA.

[25] Gkekas I., Katsamakas S., Mylonas S., Fotopoulou T., Magoulas G.Ε., Tenchiu A.C., Dimitriou M., Axenopoulos A., Rossopoulou N., Kostova S. (2024). AI Promoted Virtual Screening, Structure-Based Hit Optimization, and Synthesis of Novel COVID-19 S-RBD Domain Inhibitors. J. Chem. Inf. Model..

[26] Gupta P., Gupta S., Sinha S., Sundaram S., Sharma V.K., Munshi A. (2023). In silico phytochemical repurposing of natural molecules as entry inhibitors against RBD of the spike protein of SARS-CoV-2 using molecular docking studies. Int. J. Comput. Biol. Drug Des..

[27] Cui Z., Wang S., Xu Y., Liu Y., Wang W. (2025). Potential of umami molecules against SARS-CoV-2 (Omicron) S-RBD/hACE2 interaction: An in-silico study. J. Future Foods.

[28] Aloufi B.H., Snoussi M., Sulieman A.M.E. (2022). Antiviral Efficacy of Selected Natural Phytochemicals against SARS-CoV-2 Spike Glycoprotein Using Structure-Based Drug Designing. Molecules.

[29] Singh R., Bhardwaj V.K., Sharma J., Kumar D., Purohit R. (2021). Identification of potential plant bioactive as SARS-CoV-2 Spike protein and human ACE2 fusion inhibitors. Comput. Biol. Med..

[30] Lan J., Ge J., Yu J., Shan S., Zhou H., Fan S., Zhang Q., Shi X., Wang Q., Zhang L. (2020). Structure of the SARS-CoV-2 spike receptor-binding domain bound to the ACE2 receptor. Nature.

[31] Rady I., Siddiqui I.A., Rady M., Mukhtar H. (2017). Melittin, a major peptide component of bee venom, and its conjugates in cancer therapy. Cancer Lett..

[32] Ceremuga M., Stela M., Janik E., Gorniak L., Synowiec E., Sliwinski T., Sitarek P., Saluk-Bijak J., Bijak M. (2020). Melittin—A Natural Peptide from Bee Venom Which Induces Apoptosis in Human Leukaemia Cells. Biomolecules.

[33] Mojsoska B., Jenssen H. (2015). Peptides and Peptidomimetics for Antimicrobial Drug Design. Pharmaceuticals.

[34] Pushpakom S., Iorio F., Eyers P.A., Escott K.J., Hopper S., Wells A., Doig A., Guilliams T., Latimer J., McNamee C. (2018). Drug repurposing: Progress, challenges and recommendations. Nat. Rev. Drug Discov..

[35] Lode H., Fassbender M., Schaberg T., Borner K., Koeppe P. (1994). Comparative Pharmacokinetics of the New Oral Cephalosporins. Drugs.

[36] Van Bambeke F.O., Van Laethem Y., Courvalin P., Tulkens P.M. (2004). Glycopeptide Antibiotics. Drugs.

[37] Pushkaran A.C., Nath En P., Melge A.R., Puthiyedath R., Mohan C.G. (2021). A phytochemical-based medication search for the SARS-CoV-2 infection by molecular docking models towards spike glycoproteins and main proteases. RSC Adv..

[38] Istifli E.S., Wang M., Yan H., Chen L., Wang Y., Li L., Zhang H., Miao L. (2023). Oxalic acid blocked the binding of spike protein from SARS-CoV-2 Delta (B.1.617.2) and Omicron (B.1.1.529) variants to human angiotensin-converting enzymes 2. PLoS ONE.

[39] Yang Y.-Y., Tseng Y.J. (2023). The evolution of the spike protein and hACE2 interface of SARS-CoV-2 omicron variants determined by hydrogen bond formation. Brief. Funct. Genom..

[40] Mannar D., Saville J.W., Zhu X., Srivastava S.S., Berezuk A.M., Tuttle K.S., Marquez A.C., Sekirov I., Subramaniam S. (2022). SARS-CoV-2 Omicron variant: Antibody evasion and cryo-EM structure of spike protein–ACE2 complex. Science.

