# Supplementary material for: Identification of Four Compounds with S-RBD-Binding and Pseudovirus Entry-Inhibitory Activity
Source: Int J Mol Sci. 2026 Sep 12;27(18):8136. doi: 10.3390/ijms27188136 (PMC13607727; doi:10.3390/ijms27188136)
Supplement: Supplementary file 1 [file ijms-27-08136-s001.zip › ijms-4484038-supplementary.pdf]

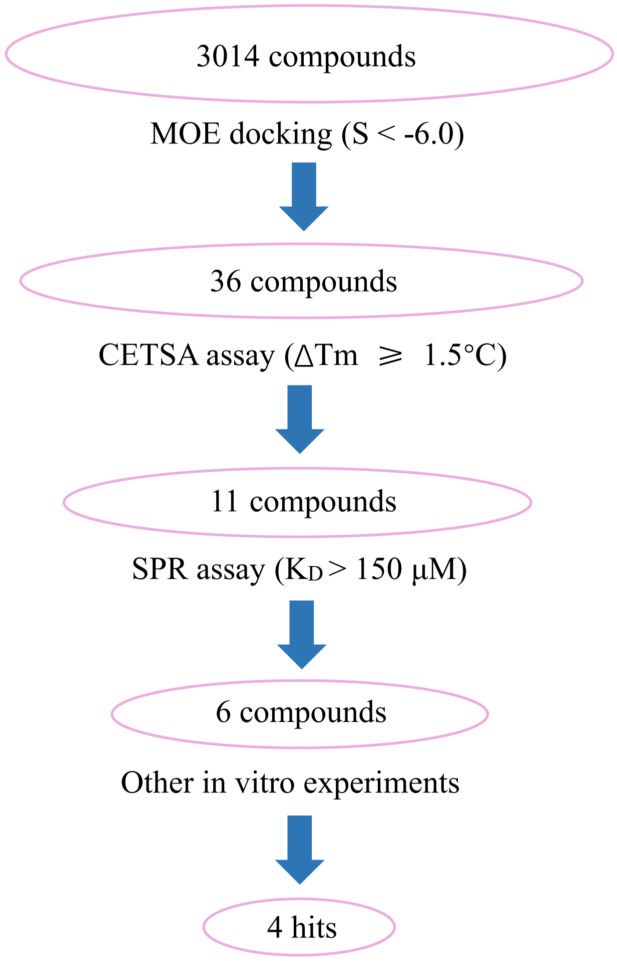

Supplementary Fig. S1. Flowchart of the compound screening funnel.
A total of 3,014 compounds were subjected to virtual screening by molecular docking; 36 candidates were selected based on a docking score < –6.0. These candidates were then evaluated by Cellular Thermal Shift Assay (CETSA); 11 of the 19 compounds tested met the positivity criterion ($\Delta Tm \geq 1.5$ °C) and were identified as candidate binders. CETSA results were interpreted as preliminary target- engagement indicators rather than definitive binding validation. Subsequently, Surface Plasmon Resonance (SPR) (KD < 150 μM) and cell- based co- immunoprecipitation (inhibition rate > 50%) were used to assess binding affinity and functional blockade, narrowing the candidate list to 5 compounds. Finally, pseudovirus entry inhibition assays against three variants (wild- type, Delta, and Omicron) identified four lead compounds with inhibitory activity across the tested variants (significant inhibition, $P < 0.05$ vs. control). SPR and functional assays served as the decisive criteria for compound prioritisation; compounds were advanced based on orthogonal confirmation of binding and activity, regardless of their CETSA profiles.

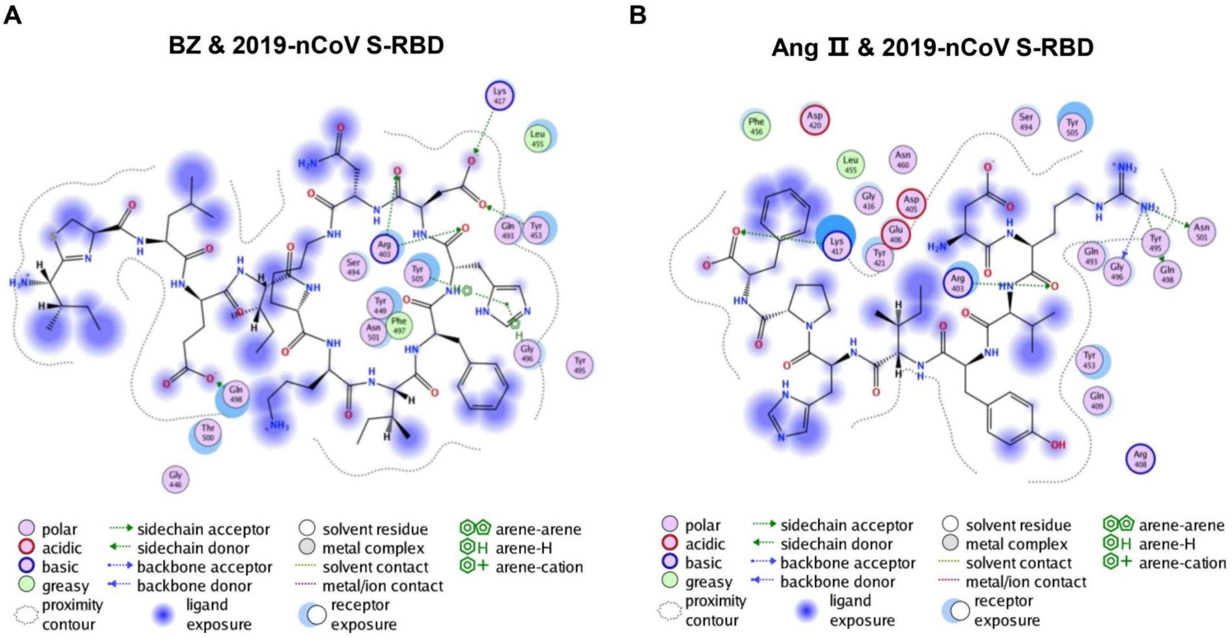

Supplementary Fig. S2. Molecular docking of two compounds with 2019-nCoV S-RBD. Two-dimensional diagram illustrates the interactions between (**A**) Bacitracin Zinc (BZ) and (**B**) Angiotensin IIHuman Acetate (Ang II) with the 2019-nCoV S-RBD.

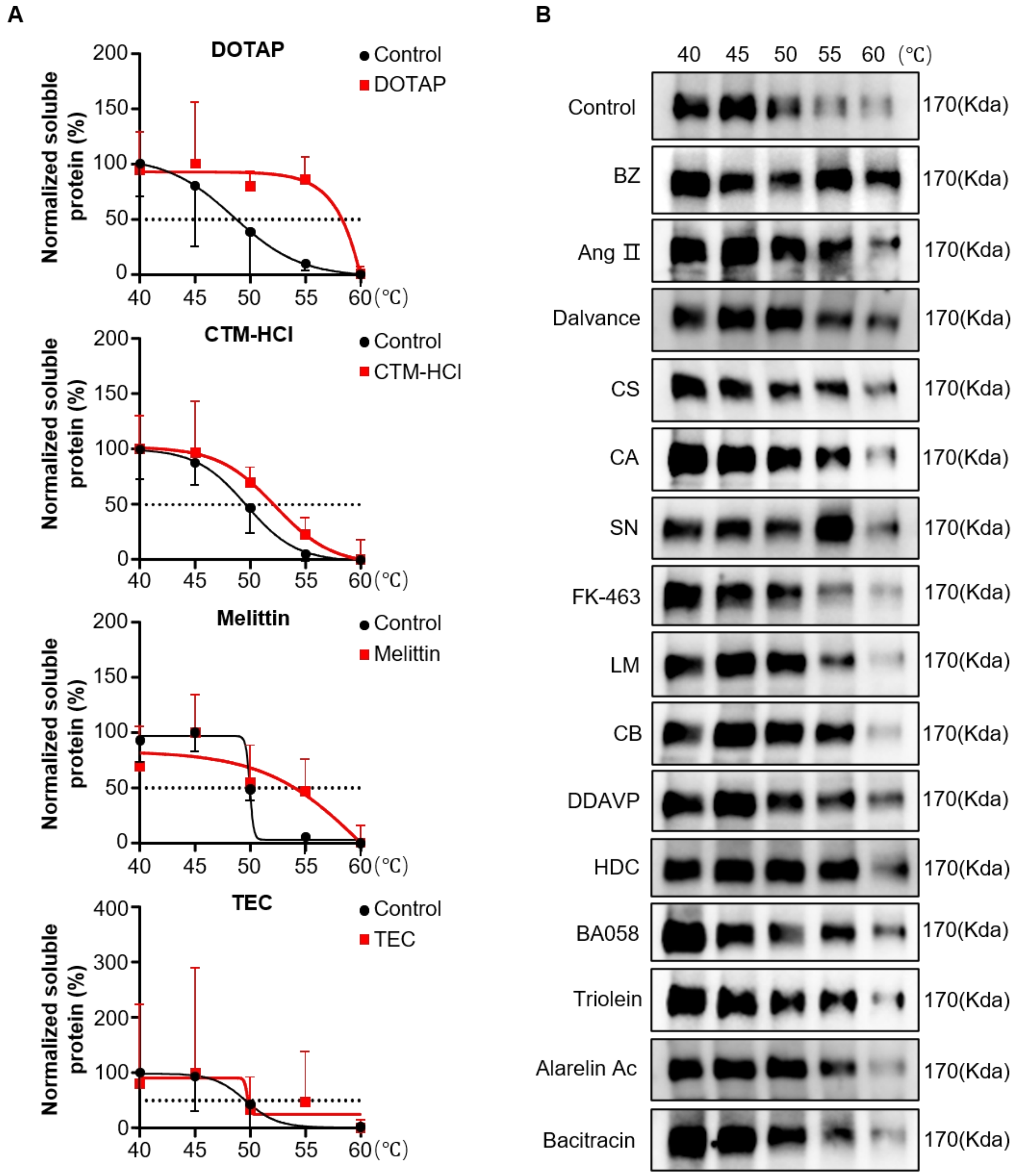

Supplementary Fig. S3. CETSA analysis.

S- protein- containing HEK293T lysates were incubated with 100 μM compounds and analyzed by Western blotting. (**A**) Melting curves (40–60°C) for the four prioritized compounds versus vehicle controls. Data are normalized to 40°C signal (mean ± SD, n=3). Dashed line: 50% reference. (**B**) Representative blots for 15 primary-screen compounds.

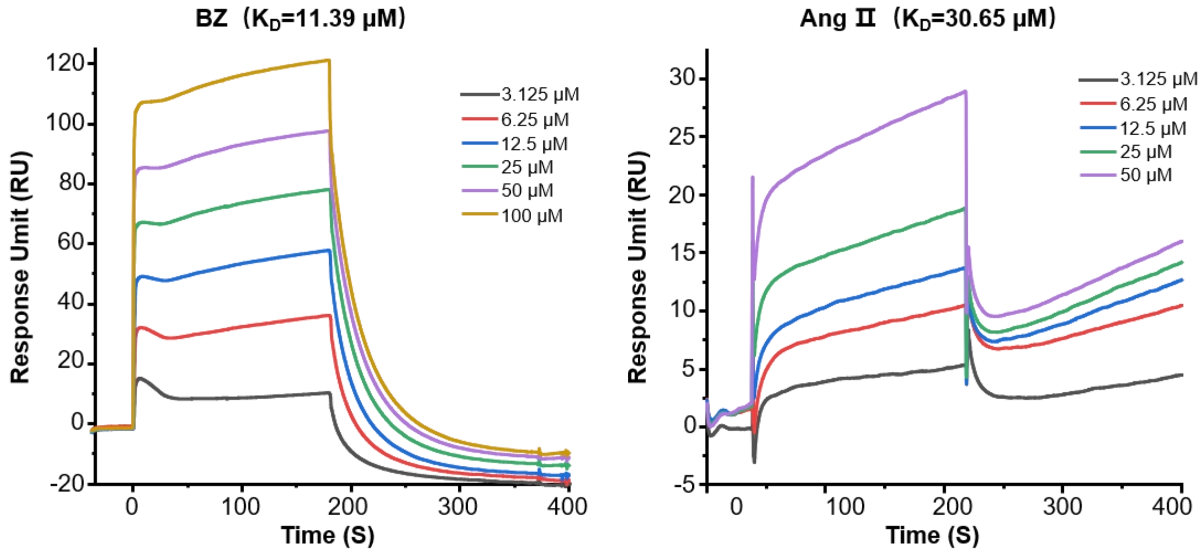

Supplementary Fig. S4. Compounds bind to 2019-nCoV S-RBD.

The 2019-nCoV S-RBD was immobilized on a CM5 sensor chip using the Biacore 8K instrument. The association and dissociation curves for two compounds are shown. The compounds were tested at various concentrations, and the K_D values were determined by fitting the data to a 1:1 binding model using Biacore Insight Evaluation Software. K_D represents the equilibrium dissociation constant.

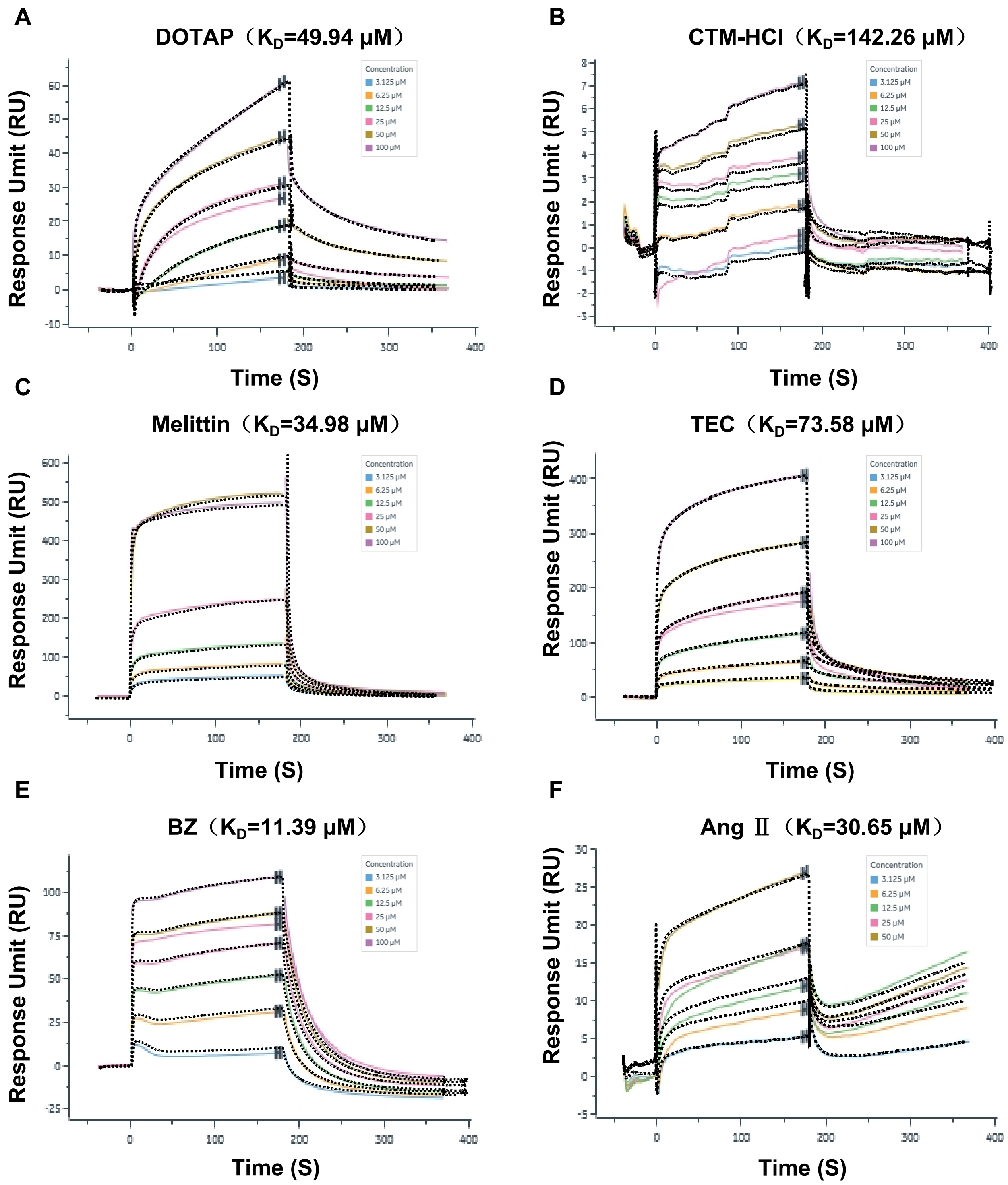

Supplementary Fig. S5. Representative SPR sensorgrams for six compounds.

Serially diluted analytes were injected over the chip surface at a flow rate of 30 μL/min. Experimental data points are shown as colored solid lines. The black dashed lines represent the global fit of the data to a 1:1 binding model, from which the kinetic parameters (k_a, k_d) and equilibrium dissociation constant (K_D) were derived. The close overlap between experimental data and fitted curves indicates a high-quality fit and supports the reliability of the calculated binding affinities. The compounds shown are: (**A**) DOTAP, (**B**) CTM-HCl, (**C**) Melittin, (**D**) TEC, (**E**) BZ, and (**F**) Ang **II**.

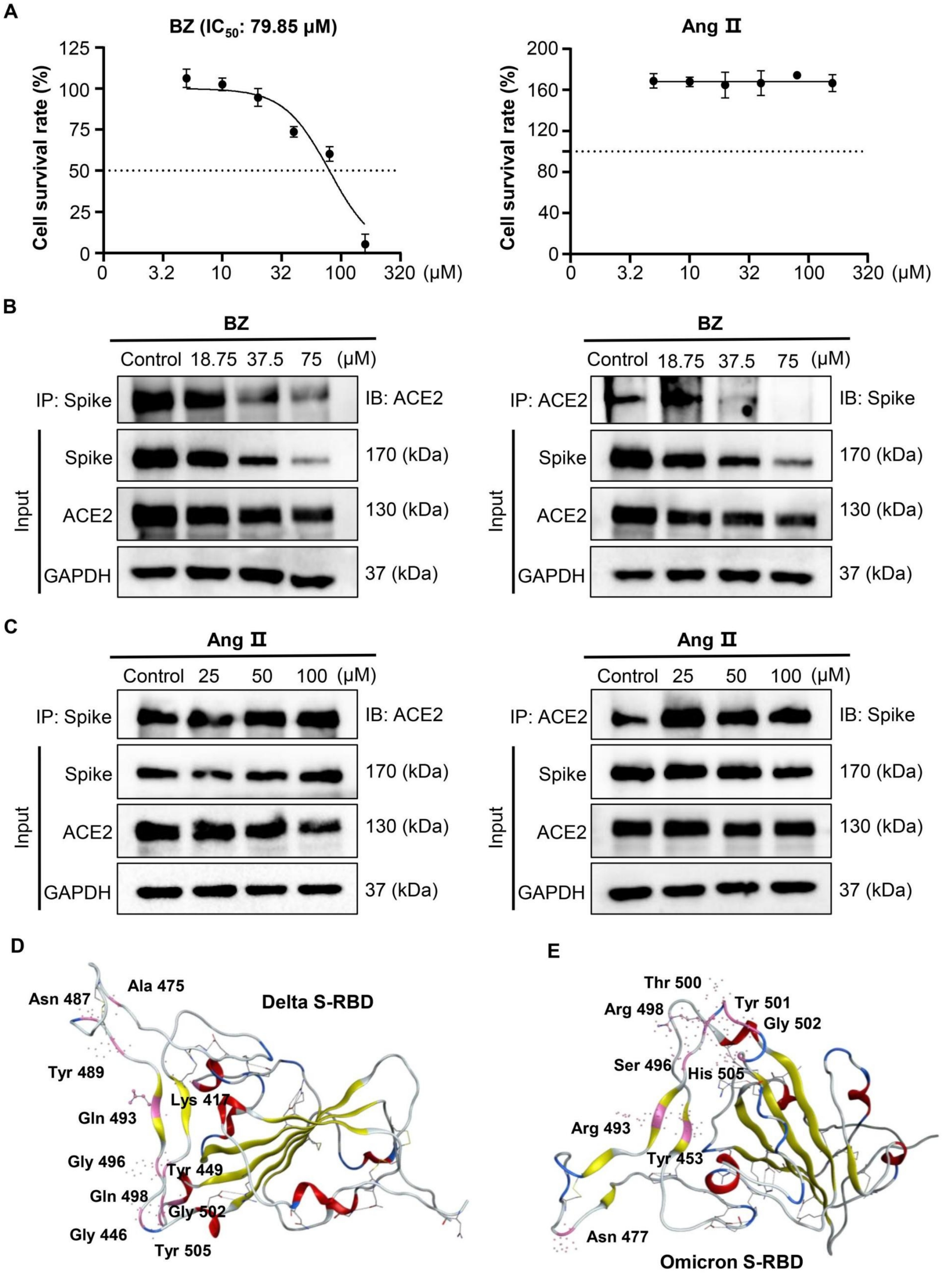

Supplementary Fig. S6.

The cytotoxicity of **(A)** BZ or Ang II was assessed in HEK293T cells, with IC_{50} values measured at 24 hours. Co-immunoprecipitation (Co-IP) assays were performed to investigate the interaction between SARS-CoV-2 Spike protein and hACE2 in HEK293T cells. Cells were treated with **(B)** BZ or **(C)** Ang II for 24 hours. Immunoprecipitation was carried out using anti-hACE2 or anti-Spike antibodies, followed by immunoblotting with antibodies against hACE2, Spike, and GAPDH. Crystal structure of the **(D)** Delta S-RBD and **(E)** Omicron S-RBD. The binding sites of S-RBD with hACE2 are shown in pink.

Supplementary Table S1. Report on residues involved in the interaction of two compounds

Compound	Ligand	Receptor (S-RBD)	Interaction	E (kcal/mol)
BZ	-COOH	Gln 498	H-acceptor	-0.7
	-CONH-	Arg 403	H-acceptor	-3.3
	-COOH	Lys 417	H-acceptor	-0.5
	-COOH	Tyr 453	H-acceptor	-2.6
	-C3H4N2	Gly 496	Π-H	-2.0
	-C3H4N2	Tyr 505	Π-H	-1.3
Ang Ⅱ	-NHC (=NH) NH:	Gly 496	H-donor	-1.4
	-NHC (=NH) NH:	Gln 498	H-donor	-2.6
	-NHC (=NH) NH:	Asn 501	H-donor	-1.5
	-COOH	Lys 417	H-acceptor	-1.0
	-CONH-	Arg 403	H-acceptor	-3.3

Supplementary Table S2. CETSA data for all tested compounds

Compound	Tm (°C)	ΔTm (°C)
Ctr (control)	49.6	–
BZ	43.4	−6.1
Ang II	55.4	+5.8
Dalvance	53.8	+4.2
CS	48.1	−1.5
CA	51.9	+2.3
SN	51.1	+1.5
FK-463	50.5	+0.9
LM	52.5	+2.9
CB	56.2	+6.6
DDAVP	48.9	−0.7
HDC	56.9	+7.3
BA058	45.3	−4.3
Triolein	55.0	+5.4
Alarelin Ac	55.1	+5.5

Supplementary Table S3. SPR Binding Parameters of the Four Lead Compounds

Compound	K_D (μM)	Mean K_D ± SD (μM)	CV (%)	χ^2	Rmax (RU)	R^2
	49.94			3.3	92.4	0.99
DOTAP		64.48 ± 20.57	31.9			
	79.02			4.3	44.1	0.91
CTM-HCl	142.26	142.26 ± N/A	N/A	2.5	16	0.97
	57.02			30658.5	2575.7	0.95*
Melittin	34.08	49.60 ± 12.25	25.5	5002.6	777.1	0.94
	56.79			22939.3	2649.4	0.94
	109.13			28.1	1408.8	0.98
TEC	122.03	101.58 ± 25.10	24.7	2.6	669.9	0.98
	73.58			53.2	682.3	0.96

*R^2 value calculated after exclusion of the 50 μM outlier.

Supplementary Table S4. Report on molecular docking scores and interacting residues for 5 compounds

Compound	Docking score (s)	Receptor		Interaction
DOTAP	-7.23	Delta	Gly 496	H-acceptor
	-7.70	Omicron	Ser 496	H-acceptor
			Arg 498	H-acceptor
			Tyr 501	Π-H
			His 505	Π-H
CTM-HCl	-7.52	Delta	Asn 501	H-donor
			Tyr 505	H-donor
			Thr 500	H-donor
			Asp 405	Ionic
			Gly 502	Π-H
			Tyr 505	Π-H
	-7.39	Omicron	Arg 403	Π-H
			Tyr 501	Π-H
Melittin	-12.13	Delta	Lys 478	H-acceptor
			Asn 481	H-acceptor
			Phe 486	H-acceptor
			Cys 488	H-donor
			Gln 498	H-donor
			Thr 500	H-donor
	-15.76	Omicron	Leu 455	H-donor
			Asn 477	H-donor
			Gly 476	H-donor
			Ser 496	H-acceptor
			His 505	Π-H
TEC	-8.62	Delta	Tyr 449	Π-H
			Leu 455	Π-H
			Asn 501	H-donor
	-9.14	Omicron	Arg 403	H-donor
			Tyr 449	H-acceptor
			Ser 496	H-acceptor
			His 505	Π-H

Supplementary Table S5. Summary of antiviral potency and therapeutic index for the four lead compounds against three SARS-CoV-2 variants

Compounds	Pseudovirus	Pseudovirus IC_{50} (μM)	Cytotoxicity CC_{50} (μM)	Selectivity Index (SI)
DOTAP	2019-nCoV	> 100	NA	ND
	Delta	>100		
	Omicron	>100		
CTM-HCl	2019-nCoV	94.60	> 100	>1.06
	Delta	>100		ND
	Omicron	>100		ND
Melittin	2019-nCoV	1.83	2.3	1.26
	Delta	1.99		1.16
	Omicron	>1.25		<1.84
TEC	2019-nCoV	> 100	> 100	ND
	Delta	>100		
	Omicron	>100		

Note: SI = CC_{50} / IC_{50}
